# Correction: Rediscovery of the enigmatic fungus-farming ant "*Mycetosoritis*" *asper* Mayr (Hymenoptera: Formicidae): Implications for taxonomy, phylogeny, and the evolution of agriculture in ants

**DOI:** 10.1371/journal.pone.0181737

**Published:** 2017-07-13

**Authors:** The PLOS ONE Staff

## Notice of Republication

This article was republished on July 3, 2017, to correct errors in the formatting that were introduced during the typesetting process. The publisher apologizes for the errors.

The Data Availability statement for this paper is incorrect. The correct statement is: New sequences generated for this study are deposited in GenBank under accession numbers KY809160–KY809180 for the fungal cultivar and KY828479–KY828592 for the ants. Nexus and tree files can be found in Dryad (http://datadryad.org/resource/doi:10.5061/dryad.64r0j).

Please download this article again to view the correct version. The originally published, uncorrected article and the republished, corrected articles are provided here for reference.

## Supporting information

S1 File Originally published, uncorrected article. (PDF)

S2 File Republished, corrected article. (PDF)
