# Supplementary material for: Correction: Rediscovery of the enigmatic fungus-farming ant "Mycetosoritis" asper Mayr (Hymenoptera: Formicidae): Implications for taxonomy, phylogeny, and the evolution of agriculture in ants
Source: PLoS One. 2017 Jul 13;12(7):e0181737. doi: 10.1371/journal.pone.0181737 (PMC5509369; doi:10.1371/journal.pone.0181737)
Supplement: S1 File — (PDF) [file pone.0181737.s001.pdf]

PLOS | ONE

RESEARCH ARTICLE

Rediscovery of the enigmatic fungus-farming ant "*Mycetosoritis*" *asper* Mayr (Hymenoptera: Formicidae): Implications for taxonomy, phylogeny, and the evolution of agriculture in ants

Jeffrey Sosa-Calvo[1,2,3¤]*, Ana Ješovnik[2,3], Heraldo L. Vasconcelos[4], Mauricio Bacci, Jr.[5], Ted R. Schultz[2]*

1 Department of Biology, University of Rochester, Rochester NY, United States of America, **2** Department of Entomology, National Museum of Natural History, Smithsonian Institution, Washington DC, United States of America, **3** Department of Entomology, University of Maryland, College Park, MD, United States of America, **4** Instituto de Biologia, Universidade Federal de Uberlândia, Uberlândia, Minas Gerais, Brazil, **5** Centro de Estudos de Insetos Sociais, Universidade Estadual Paulista, Rio Claro, São Paulo, Brazil

¤ Current address: Center for Social Insect Research, School of Life Sciences, Arizona State University, Tempe, AZ, United States of America
* schultzt@si.edu (TRS); jsosacalvo@gmail.com (JSC)

Check for updates

OPEN ACCESS

Citation: Sosa-Calvo J, Ješovnik A, Vasconcelos HL, Bacci M, Jr., Schultz TR (2017) Rediscovery of the enigmatic fungus-farming ant "*Mycetosoritis*" *asper* Mayr (Hymenoptera: Formicidae): Implications for taxonomy, phylogeny, and the evolution of agriculture in ants. PLoS ONE 12(5): e0176498. https://doi.org/10.1371/journal.pone.0176498

Editor: Fabio S. Nascimento, Universidade de Sao Paulo Faculdade de Filosofia Ciencias e Letras de Ribeirao Preto, BRAZIL

Received: January 17, 2017

Accepted: April 11, 2017

Published: May 10, 2017

Copyright: This is an open access article, free of all copyright, and may be freely reproduced, distributed, transmitted, modified, built upon, or otherwise used by anyone for any lawful purpose. The work is made available under the Creative Commons CC0 public domain dedication.

Data Availability Statement: New sequences generated for this study are deposited in GenBank under accession numbers KY809160–KY809180 for the fungal cultivar and KY828479–KY828592 for the ants. Nexus and tree files can be found in Treebase (http://purl.org/phylo/treebase/phylows/study/TB2:S20854).

Abstract

We report the rediscovery of the exceedingly rarely collected and enigmatic fungus-farming ant species *Mycetosoritis asper*. Since the description of the type specimen in 1887, only four additional specimens are known to have been added to the world's insect collections. Its biology is entirely unknown and its phylogenetic position within the fungus-farming ants has remained puzzling due to its aberrant morphology. In 2014 we excavated and collected twenty-one colonies of *M*. *asper* in the Floresta Nacional de Chapecó in Santa Catarina, Brazil. We describe here for the first time the male and larva of the species and complement the previous descriptions of both the queen and the worker. We describe, also for the first time, *M*. *asper* biology, nest architecture, and colony demographics, and identify its fungal cultivar. Molecular phylogenetic analyses indicate that both *M*. *asper* and *M*. *clorindae* are members of the genus *Cyphomyrmex*, which we show to be paraphyletic as currently defined. More precisely, *M*. *asper* is a member of the *Cyphomyrmex strigatus* group, which we also show to be paraphyletic with respect to the genus *Mycetophylax*. Based on these results, and in the interest of taxonomic stability, we transfer the species *M*. *asper*, *M*. *clorindae*, and all members of the *C*. *strigatus* group to the genus *Mycetophylax*, the oldest available name for this clade. Based on ITS sequence data, *Mycetophylax asper* practices lower agriculture, cultivating a fungal species that belongs to lower-attine fungal Clade 2, subclade F.

Funding: T.R.S., and J.S.-C, were partially supported by National Science Foundation (NSF) grants DEB-1456964 and DEB- 0949689 and the National Museum of Natural History (NMNH) Small Grants program; T.R.S. by the Smithsonian Institution Scholarly Studies Program; J.S.-C. and A.J. by NMNH Peter Buck Predoctoral Fellowships and Max and Vera Britton Environmental Science Awards (Cosmos Club Foundation); M.B. by the Fundação de Amparo à Pesquisa do Estado de São Paulo (FAPESP; 2011/50226-0) and CNPq (311562/2012-4 and 487639/2012-0), and H.L.V. by the Brazilian Council of Research and Scientific Development (CNPq grant 302588/2015-9).

Competing interests: The authors have declared that no competing interest exist.

Introduction

Although many species are easily assignable to existing genera based on morphology, some are not. Rather than assign them the status of "*incertae sedis*," taxonomists often group such species into so-called "dust-bin" genera erected for species of uncertain relationship, even when the species within such genera bear little resemblance to one another. Yet numerous studies have demonstrated that such phylogenetically isolated species may be especially important for understanding deeper relationships of genera, tribes, and subfamilies [1–8]. Including those species in phylogenetic analyses has significant effects on topology, ancestral character-state reconstruction, divergence-time estimation, and inferences of evolutionary rates [9–11].

Within the fungus-farming ants (Myrmicinae, Attini, Attina; hereafter “attine” ants [12–14]), the genus *Mycetosoritis* Wheeler has historically served as a "dust-bin" genus. Since its creation, *Mycetosoritis* has been regarded as a “degenerate and simplified *Trachymyrmex* or an aberrant *Cyphomyrmex*” ([15]: 716) or as transitional between the genera *Cyphomyrmex* Mayr and *Trachymyrmex* Forel [16–18]. The genus was first established by Wheeler in 1907 [15] as a subgenus of *Atta* Fabricius to accommodate the previously described species *Cyphomyrmex asper* (Mayr) and the newly described *M. hartmanni* (Wheeler). Later, in 1913 and in 1922, Emery ([19]: 251; [20]: 343–344) transferred *Mycetosoritis* to *Cyphomyrmex*, which in his definition also contained *Trachymyrmex* (as a subgenus). In 1922, Wheeler ([21]: 669) transferred the subgenus *Mycetosoritis* to *Trachymyrmex*. In 1949, Kusnezov [22] described the species *Cyphomyrmex* (*Mycetosoritis*) *clorindae* from Argentina, pointing out its resemblance to other members of *Cyphomyrmex* except for its erect pilosity, which contrasted with the appressed and fine, scale-like pilosity of *Cyphomyrmex s. str*. In addition, Kusnezov [22] suggested that the morphological characters of *clorindae* failed to fully agree with those of the genus *Mycetosoritis*. As a result, in 1950, Creighton ([16]: 317–318) elevated *Mycetosoritis* to genus status, arguing that “to include such an obviously transitional species in either genus [*Cyphomyrmex* and *Trachymyrmex*] weakens the distinctions by which they may be separated.” As part of his revision of the *Cyphomyrmex strigatus* group in 1964, Kempf [17] acknowledged that the species *Mycetosoritis asper* [23] and *Mycetosoritis clorindae* [22] are rather different from *Mycetosoritis hartmanni* and that both species share some affinities with members of the *C. strigatus* group, including the presence of a well-developed antennal scrobe. Kempf, however, argued against including those species within *Cyphomyrmex* "as a provisional and temporary measure" due to the presence of erect hairs in both species (hairs are appressed and scale-like in *Cyphomyrmex*) and the absence of males for comparison.

Since Creighton [16] elevated *Mycetosoritis* to genus status, two additional species have been described based on workers: *M. explicatus* from Brazil in 1968 [18] and *M. vinsoni* from Costa Rica in 1998 [24]. In his description of *M. explicatus*, Kempf [18] drew attention to the high variability of the genus *Mycetosoritis* as defined by Emery [20] and discussed the difficulty of placing *M. explicatus* into either *Cyphomyrmex* or *Mycetosoritis*, arguing that it shares character states with both, but he ultimately chose the latter based on pilosity. Most recently, the monophyly of *Mycetosoritis* has been questioned [25].

In this study, we focus on the species *Mycetosoritis asper*, which hitherto has remained exceedingly rare in insect collections. To date, the species is known in the literature from the type specimen, an alate queen from Santa Catarina described by Mayr in 1887 [23], and a single worker subsequently collected in Puerto Piray, Misiones, Argentina, and described by Emery in 1906 [26]. Since then, only three additional workers have been collected (one in 1957 and two in 1999) in Santa Catarina, Brazil. These last three records remain unpublished and the biology of the species remains, heretofore, entirely unknown. In an attempt to clarify the phylogenetic position of this species within the fungus-farming ants, as well as to learn more

about its biology, we conducted field research in the Floresta Nacional de Chapecó, Santa Catarina, Brazil. Here, for the first time, we: (i) describe the male and larva of *Mycetosoritis asper*, (ii) document its nest architecture and demographics based on twenty-one colonies collected, (iii) report on the phylogenetic position of *M*. *asper* within the subtribe Attina by generating new DNA sequences and conducting multilocus phylogenetic analyses, and (iv) report on the identity of its fungal cultivar and the agricultural system to which it belongs.

Materials and methods

Field observations and nest excavations

Field work was conducted 18–21 October 2014 in the Floresta Nacional de Chapecó (henceforth FLONA Chapecó), located between the municipalities of Guatambú and Chapecó in the west of the state of Santa Catarina, Brazil. The FLONA Chapecó is divided into three zones or Glebas: Glebas I and III are located in the municipality of Guatambú, whereas Gleba II is located in the municipality of Chapecó [27, 28]. The FLONA Chapecó contains remnants of Atlantic Forest, including *Araucaria angustifolia*, as well as plantations of *A*. *angustifolia*, pines (*Pinus elliottii*, *P*. *taeda*), and *Eucalyptus* species. The FLONA Chapecó is surrounded by intensively disturbed habitat used mostly for agriculture, pasture, and silviculture [28]. The most common types of soil in the FLONA Chapecó are Cambisols and Latosols. For more information regarding the FLONA Chapecó see ICMbio [28]. The FLONA Chapecó Gleba I, where we located twenty-one nests of *Mycetosoritis asper* (at 27.10306˚ S 52.77898˚ W, elevation 595–601 m above sea level), has an estimated area of 1300 ha and is considered a remnant of Atlantic Forest (containing mixed ombrophilous and seasonal deciduous forests), with a mean annual rainfall of 2007 mm and a mean annual temperature of 22˚ C [27–29].

Foraging activity by workers of *Mycetosoritis asper* was observed during the day. Foragers were located by baiting the area with Cream of Rice® cereal spread generously on the ground. The workers carrying the bait were then followed to their nest entrances. Nest entrances were marked with flagging and dug up when a substantial number of nest entrances had been located. Nests were excavated following Schultz [30], Rabeling et al., [31], and Sosa-Calvo et al., [32]. The twenty-one excavated colonies were transferred, using flame-sterilized forceps and spoons, from their subterranean chambers into plastic nest boxes containing a layer of plaster at the bottom, which was saturated with water [32]. Eleven of the twenty-one colonies collected are still maintained alive in artificial nest boxes in the AntLab at the Smithsonian Institution in Washington, DC (Table 1).

Table 1. Nest measurements and colony demographics of 21 excavated nests of *Mycetophylax asper* in Floresta Nacional de Chapecó (FN-Chapecó).

				CHAMBER DIMENSIONS (cm)			Field notes	Laboratory notes*
**Nest**	**Coll. Code**	**Date**	**Depth (cm)**	**Height**	**Width**	**Depth**		
**1**	AJ141018–01	Oct 18, 2014	44	5	5	10	Large garden, several workers and brood present, **queen present**	Produced mostly gynes and a single male. Production of reproductives from mid Feb to mid Mar. Colony expired Nov 2015
**2**	AJ141018–02	Oct 18, 2014	48	6	6	6	**Queen present**	Colony still **alive** in laboratory. Did not produce reproductives

(*Continued*)

Table 1. (*Continued*)

				CHAMBER DIMENSIONS (cm)			Field notes	Laboratory notes*
Nest	Coll. Code	Date	Depth (cm)	Height	Width	Depth		
3	AJ141018–03	Oct 18, 2014	36	5	5	6	Large garden, several workers and brood present, **queen present**	Colony still **alive** in laboratory. Did not produce reproductives
4	AJ141018–04	Oct 18, 2014	39	4.5	7	9	Large garden, partially yellowish with some parts greenish, several workers and brood present, **queen present**	Produced 7 alate gynes and 3 males. Production of reproductives from mid Feb to beginning of Mar. Colony still **alive** in laboratory
5	AJ141018–05	Oct 18, 2014	42	5	5	6	Large garden, several workers and brood present, **queen present**	Colony still **alive** in laboratory. Did not produce reproductives
6	AJ141018–08	Oct 18, 2014	30	5	8	6	Chamber was accidentally opened from the top, garden partially ruined. Large garden, several workers and brood present, **queen present**	Did not produce reproductives. Colony expired Jul 2016
7	AJ141018–09	Oct 18, 2014	40	5	8	7	Large garden, several workers and brood present, **queen present**	Did not produce reproductives. Colony expired Feb 2016
8	AJ141018–11	Oct 18, 2014	29	6	5	7	Large garden, partially yellowish with some parts greenish and a large pellet of wet dirt/refuse located on chamber floor, several workers and brood present, **queen present**	Colony still **alive** in laboratory. Did not produce reproductives
9	AJ141020–01	Oct 20, 2014	50	3	3	3	Very small chamber. Small garden, few workers present, **queen present**	Did not produce reproductives. Colony expired Feb 2016
10	AJ141020–02	Oct 20, 2014	47	6	7	7	Large garden, large pellet of wet dirt/refuse located on chamber floor, several workers and brood present, **queen present**	Produced only alate gynes. Production of reproductives from mid Feb to mid Mar. Colony still **alive** in laboratory
11	JSC141018–01	Oct 18, 2014	60	7	5.7	4.5	Single chamber containing small compact garden, several workers present, **queen present**	Colony still **alive** in laboratory. Did not produce reproductives
12	JSC141018–04	Oct 18, 2014	65	4	7	7	Chamber found by accident and destroyed during excavation, **queen present**	Colony still **alive** in laboratory. Did not produce reproductives
13	JSC141018–08	Oct 18, 2014	9	2	3	1	Incipient nest, small chamber, few workers present, **queen present**	Did not produce reproductives. Colony expired Mar 2015
14	JSC141018–10	Oct 18, 2014	34	2.5	3.5	1.5	Very small chamber. Small garden, few workers present, **queen present**	Did not produce reproductives. Colony expired May 2016
15	JSC141020–01	Oct 20, 2014	48	6	7	5.5	Large chamber with large fungus garden. Chamber convex on top and somewhat flattened on bottom. A large pellet of wet dirt/refuse located on chamber floor, several workers and brood present, **queen present**	Produced only alate gynes. Production of reproductives from mid Feb to mid Mar. Colony still **alive** in laboratory
16	JSC141020–03	Oct 20, 2014	40	6	6	6	Large chamber with large fungus garden. Chamber convex on top and somewhat flattened on bottom. A large pellet of wet dirt/refuse located on chamber floor, several workers and brood present, **queen present**	Produced only males. Production of reproductives from mid Mar to mid Apr. Colony still **alive** in laboratory
17	JSC141020–04	Oct 20, 2014	65	4	6	5	Large garden, several workers and brood present, **queen present**	Produced only alate gynes. Production of reproductives only in Feb. Colony still **alive** in laboratory

(*Continued*)

Table 1. (*Continued*)

				CHAMBER DIMENSIONS (cm)			Field notes	Laboratory notes*
**Nest**	**Coll. Code**	**Date**	**Depth (cm)**	**Height**	**Width**	**Depth**		
**18**	JSC141021–01	Oct 21, 2014	10	1	1.5	0.5	Incipient nest, very small chamber, workers not observed, **queen present**	Collected into alcohol at time of excavation
**19**	JSC141021–02	Oct 21, 2014	50	6	7.5	6.5	Large chamber with large fungus garden. Fungus garden hanging from ceiling of chamber, probably attached to rootlets. A large pellet of wet dirt/refuse located on chamber floor, several workers and brood present, **queen present**	Produced 33 alate gynes and 3 males. Production of reproductives from beginning of Feb to mid of Mar. Colony expired Apr 2016
**20**	JSC141021–04	Oct 21, 2014	43.5	6	6	6	Large chamber with large fungus garden. Chamber convex on top and somewhat flattened on bottom. A large pellet of wet dirt/refuse located on chamber floor, several workers and brood present, **queen present**	Produced 9 alate gynes and 3 males. Production of reproductives from end of Jan to mid of Feb. Colony expired Apr 2015
**21**	TRS141017–01	Oct 17, 2014	52	5	6	6	Large garden, several workers present, brood not observed, **queen present**	Colony still **alive** in laboratory. Did not produce reproductives

* Observations conducted from Nov 2014 to October 2015 when feeding colonies (three times a week). Numbers in **bold** in the "Nest" column indicate colonies that remain alive in the Smithsonian AntLab. In addition to males and gynes, lab colonies also produced workers, but worker numbers were not recorded.

https://doi.org/10.1371/journal.pone.0176498.t001

Fungus garden fragments were isolated and axenically cultured on PDA (potato dextrose agar) medium with three antibiotics (Penicillin G, Streptomycin sulfate, and Chloramphenicol) in the Smithsonian Institution AntLab. Mycelia were transferred from agarose to PDA liquid broth medium with no antibiotics and cultured at 30°C under constant agitation in a New Brunswick Scientific Series 25 Incubator Shaker, after which the tissues were filtered, lyophilized, and placed into cryo-storage for later DNA extraction.

Nest architecture was recorded, photographed, and measured following Sosa-Calvo et al., ([32]; page 307) Fungus garden fragments and a subset of workers were preserved in 95% ethanol in the field. Fungal vouchers are deposited in the USNM and ant vouchers are deposited in the insect collections of the following institutions:

**CRC**	C. Rabeling Collection, Arizona State University, Tempe, AZ, U.S.A.
**DZUP**	Coleção Entomológica "Padre Jesus Santiago Moure," Departamento de Zoologia, Universidade Federal do Paraná, Curitiba, Paraná, Brazil.
**ICN**	Instituto de Ciencias Naturales, Universidad Nacional de Colombia, Bogotá, DC, Colombia.
**MPEG**	Museu Paraense 'Emilio Goeldi,' Belém, Pará, Brazil.
**MZSP**	Museu de Zoologia, Universidade de São Paulo, São Paulo, Brazil.
**MBC-UFU**	Museu de Biodiversidade do Cerrado, Universidade Federal de Uberlândia, Uberlândia, Minas Gerais, Brazil.
**USNM**	United States National Museum of Natural History, Washington, DC, U.S.A.

https://doi.org/10.1371/journal.pone.0176498.t002

Material examined

In addition to the material collected by us in the FLONA Chapecó, we examined the type specimen, a dealate queen from Santa Catarina, Brazil, described by Mayr [23], and a worker from Puerto Piray, Misiones, Argentina, described by Emery [26], but in that publication erroneously recorded as collected in Chubut (see below). Other material examined includes a single worker collected in 1957 by F. Plaumann in Chapecó, Santa Catarina, and two workers collected in 1999 by Rogerio da Silva in a Winkler sample in Seara, Santa Catarina. These specimens are deposited in the following institutions:

**MSNG**	Museo Civico di Storia Naturale "Giacomo Doria," Genoa, Italy.
**MZSP**	Museu de Zoologia, Universidade de São Paulo, São Paulo, Brazil.
**NHMW**	Naturhistorisches Museum, Wien, Austria.

https://doi.org/10.1371/journal.pone.0176498.t003

Morphological measurements and specimen preparation

All measurements were taken to the nearest 0.001 mm and, unless otherwise noted, are in millimeters. Composite images were generated at the USNM Ant Lab using a JVC KY–F75U digital camera mounted on a Leica Z16 APO stereomicroscope attached to a Dell Optiplex GX620 computer. Composite images were assembled using Auto-Montage Pro® (Version 5.03.0061 BETA) software (Synoptics Ltd.). Wings of males and queens were removed from the left side of the specimen, placed on microscope slides with Euparal mounting medium, and covered with a circular cover glass. The slides were labeled with the name of the species, sex, country, and locality of collection, and the unique USNMENT number of the specimen to which the wings belong.

The larvae were dehydrated sequentially through a series of ethanol concentrations to 100% absolute and then critical-point dried in a Balzers CPD–030 using liquid CO_2 at the Scanning Electron Microscopy (SEM) Lab in the SI–NMNH and mounted on aluminum stubs. The prepared larvae, as well as a worker, a queen, and a male, were sputter-coated with 60:40 wt% gold: palladium alloy on a Cressington Scientific 108 auto/SE sputter coater to a thickness of 20–25 nm. Scanning Electron Micrographs (SEMs) of these specimens were generated using a Philips XL–30 ESEM with Lanthanum Hexaboride (LaB6) source and with a backscatter detector.

Measurements, indices, abbreviations, and morphological terminology follow longstanding standard protocols [7, 13, 33–38] and literature cited therein, with modifications where noted. All images were edited using Adobe Photoshop CC 2015.0.1 (Version 20150722.r.168 x64) (Adobe Inc.). The following abbreviations are used in the description: w = worker, dq = dealate queen, m = male.

Anatomical abbreviations are as follows:

Permits

Permits to conduct field work were issued to JSC, AJ, HLV, and TRS by the Conselho Nacional de Desenvolvimento Científico e Tecnológico (CNPq; Processo EXC 039/07; Portarias 267, 359) and the Instituto Chico Mendes de Conservação da Biodiversidade (ICMBio; Permits 14789–1, 14789–2, 14789–9). Permits to export and import live ant colonies were issued to TRS and MB by the Instituto Brasileiro do Meio Ambiente e dos Recursos Naturais Renováveis

EL	*Eye Length*: in profile view, the maximum diameter of the eye measured from the dorsal margin to the ventral margin. This measurement was usually taken from the right eye from the observer point of view.
FLD	*Frontal Lobe Distance*: in full-face view, the maximum horizontal distance between the outer borders of the frontal lobes.
GL	*Gaster Length*: in profile, the length of the gaster from the anteriormost point of first gastral segment (fourth abdominal segment) to the posteriormost point of the last segment.
HFL	*Hind Femur Length*: in most appropriate view, the maximum length of the hind femur.
HTL	*Hind Tarsomere I Length*: in most appropriate view, the maximum length of the hind tarsomere I.
HL	*Head Length*: in full-face view, the maximum vertical distance from the posteriormost margin of the head to the midpoint of the anterior clypeal margin (clypeal apron), excluding the mandibles.
HW	*Head Width*: in full-face view, the maximum horizontal width of the cephalic capsule excluding the eyes.
ML	*Mandible Length*: in full-face view, the maximum diagonal-line distance from the base of the external mandibular insertion to the apical tooth. When mandibles were closed, the mandible on top was measured. When mandibles were open, then the left mandible was measured.
MSLI	*Median Clypeal Seta Length I*: in full-face view, the maximum length of the unpaired median clypeal seta from its point of origin on the clypeal apron to the tip (apex) of the seta.
MSLII	*Median Clypeal Seta Length II*: in full-face view, the maximum length of the unpaired median clypeal seta from the point where it surpasses the anterior margin of clypeal apron to the tip (apex) of the seta.
PL	*Petiole Length*: in lateral view, the straight-line distance from the posteriormost margin of the petiole to the posteriormost margin of the metapleural lobe.
PPL	*Postpetiole Length*: in lateral view, the maximum length of the postpetiole.
PPW	*Postpetiole Width*: in dorsal view, the maximum horizontal width of the postpetiole.
SL	*Scape Length*: in full-face view, the maximum length of the scape excluding the basal condyle.
TL	*Total Length*: HL+ML+WL+PL+PPL+GL.
WL	*Weber's Length*: in lateral view, the diagonal length of the mesosoma as measured from the anteriormost dorsal extent of the pronotum to the posteriormost ventral angle of the propodeum.
CI	*Cephalic Index*: (HW/HL)*100.
FLI	*Frontal Lobes Index*: (FLD/HW)*100.
MI	*Mandibular Index*: (ML/HL)*100.
MSI	*Median Seta Index*: (MSL/HL)*100.
OI	*Ocular Index*: (EL/HW)*100.
PPI	*Postpetiole Index*: (PPW/PPL)*100.
RFLDI	*Relative Frontal Lobe Distance Index I*: (FLD/HL)*100.
RFLDII	*Relative Frontal Lobe Distance Index II*: (FLD/HW)*100.
SI	*Scape Index*: (SL/HW)*100.

https://doi.org/10.1371/journal.pone.0176498.t004

(IBAMA; live export Permit 14BR015572/DF) and to TRS by the USDA APHIS PPQ (Permit to Move Live Plant Pests, Noxious Weeds, and Soil P526P-14-01931).

Molecular phylogenetics

Ant and fungal cultivar DNA extraction, amplification, and sequencing were conducted at the Laboratories of Analytical Biology (LAB) at the National Museum of Natural History, Smithsonian Institution, Washington, DC. Genomic DNA was extracted using the Qiagen DNEasy Blood and Tissue kit (Quiagen, Inc.) for the ants and the Plant DNeasy kit (Quiagen, Inc.) for the fungus. For the ants, five nuclear protein-coding genes (EF1α-F1, EF1α-F2, wg, LW Rh, and TOP1) were amplified and sequenced following the methodology outlined in previous studies [12, 39, 40]. For the fungal cultivar, a ribosomal gene fragment, *internal transcribed*

spacer (ITS), was amplified and sequenced following [41–43]. New sequences generated for this study are deposited in GenBank under accession numbers KY809160–KY809180 for the fungal cultivar and KY828479–KY828592 for the ants. Nexus and tree files can be found in Treebase (http://purl.org/phylo/treebase/phylows/study/TB2:S20864).

Ant DNA sequences, consisting of ~3.3 kbp, were added to the aligned data set of Schultz and Brady [39] and Sosa-Calvo et al., [7] and aligned first by eye in Mesquite [44] and subsequently by using MAFFT v7.017 [45–47] as implemented in Geneious R9 v8.1.8 [48]. Data were partitioned and modeled using the program PartitionFinder v1.1.0 [49] under the Bayesian Information Criterion (BIC) with 15 data blocks consisting of the first, second, and third codon positions of each of the five gene fragments and with a user tree resulting from an unpartitioned maximum-likelihood best-tree analysis conducted in RAxML v.8.2 [50]. The eight partitions and models identified by PartitionFinder were employed in Bayesian analyses using MrBayes 3.2.2 [51] with nucmodel = 4by4, nruns = 2, nchains = 8, samplefreq = 1000, and 20 million generations, with a burn-in of 2 million generations. To address known problems with branch-length estimation in MrBayes [11, 52–56], we set brlenspr = unconstrained: Exp (100). Burn-in, convergence, and stationarity were assessed using Tracer, version 1.5 [57], by examining potential scale reduction factor values and.stat output files in MrBayes, and by using Bayes factor comparisons of harmonic-mean marginal likelihoods of pairs of runs with standard error estimated using 1,000 bootstrap pseudoreplicates in Tracer 1.5 [57], which employs the weighted likelihood bootstrap estimator of Newton and Raftery [58] as modified by Suchard et al., [59].

Fungal ITS sequences for *M. asper* were added to a preexisting data set of agaricaceous (Basidiomycota: Agaricales: Agaricaceae: Leucocoprineae) ant-associated and free-living fungi and aligned in MAFFT, producing a matrix consisting of 506 taxa and 1281 characters, including indels. Data were partitioned and modeled using the program PartitionFinder 2.0 under the kmeans algorithm [60], which does not require initial data blocks, and the corrected Akaike information criterion (AICc). The two partitions and models identified by PartitonFinder were employed in Bayesian analyses using MrBayes 3.2.2 as described above for the ant phylogenetic analyses, except that burn-in was set to 5 million generations.

Results and discussion

Systematics

Etymology of *"Mycetosoritis.* Despite a tradition of treating it as feminine, the genus name "*Mycetosoritis*" should be regarded instead as masculine and should be combined with masculine-form adjectives such as *"asper"* and "*explicatus*." W. M. Wheeler [15] originally described *Mycetosoritis* as a subgenus of *Atta*. A rereading of Wheeler's [15] description of *Mycetosoritis hartmanni* provides no guidance on the gender of *Mycetosoritis* because (i) "*hartmanni*" is genitive and (ii) the adjective "*aspera*," which Wheeler uses when he transfers the former *Cyphomyrmex asper* into *Atta* (subgenus *Mycetosoritis)*, matches the (feminine) gender of *Atta*, the genus name, and not that of *Mycetosoritis*, the subgenus name. Because there is thus no indication of gender in the original description of *Mycetosoritis*, Section 30.1.4.2 of the International Code of Zoological Nomenclature [61] applies, requiring the use of the masculine gender for genus-group names with endings of ambiguous gender for which the author did not indicate the gender either explicitly or via a clear adjectival species-group name. The assertion by G. Wheeler [62] that the second part of *"Mycetosoritis"* is derived from "Sorîtis," an alternate name for the Greek goddess Ceres, is not, even if true, sufficient to supersede the ICZN rule. Wheeler's assertion is, moreover, unsupported based on the opinion (pers. comm.) of E. Adler, Associate Professor of Classics, University of Maryland, College Park, who (i)

confirmed that the noun ending *"-is"* can be masculine or feminine and (ii) could find no reference to a god or goddess "Sorîtis" in *The Oxford Latin Dictionary* [63] or in Liddell and Scott's *Greek-English Lexicon* [64].

***Mycetophylax* Emery (1913).** he multilocus analyses presented here (Fig 1), as well as phylogenomic analyses of 950 UCE loci [14], indicate with strong support that the species *Mycetosoritis asper* and *Mycetosoritis clorindae* are members of the genus *Cyphomyrmex* as currently defined and, more specifically, of the *C. strigatus* group. Both data sets further indicate that, as currently defined, the genus *Cyphomyrmex* is paraphyletic with respect to *Mycetophylax*, *Mycetagroicus*, and the higher Attina, and that the *C. strigatus* group, as currently defined, is paraphyletic with respect to the species *Mycetophylax conformis*, *Mycetophylax morschi*, and *Mycetophylax simplex*, which are derived members of the clade containing the *C. strigatus* group. Based on these results, and in the interest of taxonomic stability, we here recognize the *C. strigatus* group as a separate genus. *Mycetophylax conformis* is the nominal species for the genus name *Mycetophylax* [19] and *"Mycetophylax"* is the oldest available species-group name for the clade containing the *C. strigatus* group. We therefore transfer all members in the *C. strigatus* group, including *Mycetosoritis asper* and *Mycetosoritis clorindae*, to the genus *Mycetophylax*.

Taxonomic synopsis of the species in *Mycetophylax* Emery. The species list presented here is modified from Kempf [17] and Klingenberg and Brandão [34]:

Mycetophylax conformis Mayr 1884[65]

Mycetophylax asper (Mayr 1887)[23] **new combination**

Mycetophylax auritus (Mayr 1887)[23] **new combination**

Mycetophylax strigatus (Mayr 1887)[23] **new combination**

Mycetophylax morschi (Emery 1888)[66]

Mycetophylax simplex (Emery 1888)[66]

Mycetophylax olitor (Forel 1893)[67] **new combination**

Mycetophylax bigibbosus (Emery 1894)[68] **new combination**

Mycetophylax lectus (Forel 1911)[69] **new combination**

Mycetophylax bruchi (Santschi 1917)[70] **new combination**

Mycetophylax faunulus (Wheeler 1925)[71] **new combination**

Mycetophylax paniscus (Wheeler 1925)[71] **new combination**

Mycetophylax daguerrei (Santschi 1933)[72] **new combination**

Mycetophylax clorindae (Kusnezov 1949)[22] **new combination**

Mycetophylax lilloanus (Kusnezov 1949)[22] **new combination**

Mycetophylax vallensis (Kusnezov 1949)[22] **new combination**

Mycetophylax nemei (Kusnezov 1957)[73] **new combination**

Mycetophylax plaumanni (Kempf 1962)[74] **new combination**

Mycetophylax occultus (Kempf 1964)[17] **new combination**

Mycetophylax andersoni (Mackay & Serna 2010)[75] **new combination**

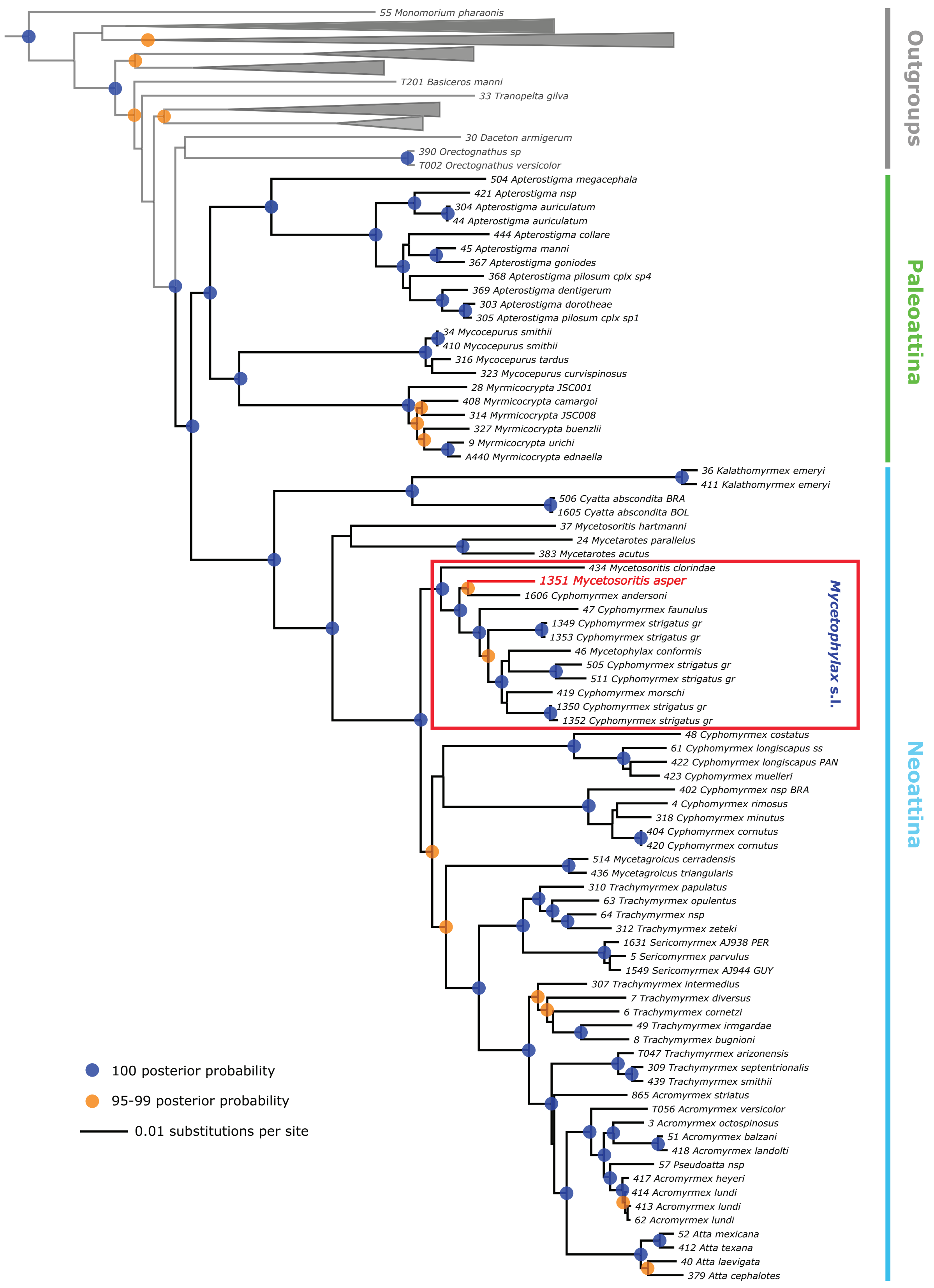

55 Monomorium pharaonis
T201 Basiceros manni
33 Tranopelta gilva
30 Daceton armigerum
390 Orectognathus sp
T002 Orectognathus versicolor
504 Apterostigma megacephala
421 Apterostigma nsp
304 Apterostigma auriculatum
44 Apterostigma auriculatum
444 Apterostigma collare
45 Apterostigma manni
367 Apterostigma goniodes
368 Apterostigma pilosum cplx sp4
369 Apterostigma dentigerum
303 Apterostigma dorotheae
305 Apterostigma pilosum cplx sp1
34 Mycocepurus smithii
410 Mycocepurus smithii
316 Mycocepurus tardus
323 Mycocepurus curvispinosus
28 Myrmicocrypta JSC001
408 Myrmicocrypta camargoi
314 Myrmicocrypta JSC008
327 Myrmicocrypta buenzlii
9 Myrmicocrypta urichi
A440 Myrmicocrypta ednaella
36 Kalathomyrmex emeryi
411 Kalathomyrmex emeryi
506 Cyatta abscondita BRA
1605 Cyatta abscondita BOL
37 Mycetosoritis hartmanni
24 Mycetarotes parallelus
383 Mycetarotes acutus
434 Mycetosoritis clorindae
1351 Mycetosoritis asper
1606 Cyphomyrmex andersoni
47 Cyphomyrmex faunulus
1349 Cyphomyrmex strigatus gr
1353 Cyphomyrmex strigatus gr
46 Mycetophylax conformis
505 Cyphomyrmex strigatus gr
511 Cyphomyrmex strigatus gr
419 Cyphomyrmex morschi
1350 Cyphomyrmex strigatus gr
1352 Cyphomyrmex strigatus gr
Mycetophylax s.l.
48 Cyphomyrmex costatus
61 Cyphomyrmex longiscapus ss
422 Cyphomyrmex longiscapus PAN
423 Cyphomyrmex muelleri
402 Cyphomyrmex nsp BRA
4 Cyphomyrmex rimosus
318 Cyphomyrmex minutus
404 Cyphomyrmex cornutus
420 Cyphomyrmex cornutus
514 Mycetagroicus cerradensis
436 Mycetagroicus triangularis
310 Trachymyrmex papulatus
63 Trachymyrmex opulentus
64 Trachymyrmex nsp
312 Trachymyrmex zeteki
1631 Sericomyrmex AJ938 PER
5 Sericomyrmex parvulus
1549 Sericomyrmex AJ944 GUY
307 Trachymyrmex intermedius
7 Trachymyrmex diversus
6 Trachymyrmex cornetzi
49 Trachymyrmex irmgardae
8 Trachymyrmex bugnioni
T047 Trachymyrmex arizonensis
309 Trachymyrmex septentrionalis
439 Trachymyrmex smithii
865 Acromyrmex striatus
T056 Acromyrmex versicolor
3 Acromyrmex octospinosus
51 Acromyrmex balzani
418 Acromyrmex landolti
57 Pseudoatta nsp
417 Acromyrmex heyeri
414 Acromyrmex lundi
413 Acromyrmex lundi
62 Acromyrmex lundi
52 Atta mexicana
412 Atta texana
40 Atta laevigata
379 Atta cephalotes
Outgroups
Paleoattina
Neoattina
100 posterior probability
95-99 posterior probability
0.01 substitutions per site

Fig 1. Phylogeny of fungus-farming ants based on Bayesian analysis of five nuclear protein-coding genes. *Mycetophylax asper* is indicated in red. Red box indicates our newly expanded definition of the genus *Mycetophylax* (see text for details).

https://doi.org/10.1371/journal.pone.0176498.g001

Mycetophylax snellingi (Mackay & Serna 2010)[75] **new combination**

Mycetophylax asper (Mayr 1887) **new combination**

Figs 2–8.

Cyphomyrmex asper Mayr 1887: 561–562, (q). Holotype alate queen: Brazil; Santa Catarina (*Hečko*). Deposited in NHMW [USNMENTNo.00923112] (examined).

Combination in *Mycetosoritis*; Emery 1924: 344. Hymenoptera. Fam. Formicidae. Subfam. Myrmicinae. [concl.]. *Genera Insectorum* 174C: 207–397.

Diagnosis. Inner margin of mandibles with 6–7 teeth; preocular carina extending posterad to cephalic corner, forming a well-developed antennal scrobe (shared with members of the former *Cyphomyrmex strigatus* group, *Mycetophylax clorindae*, and *Mycetophylax morschi*, but not with *Mycetophylax conformis* and *Mycetophylax simplex*, in which the scrobe is secondarily reduced); anterior margin of antennal scape with a broad carina, gradually expanding apically; body covered with short, erect, simple hairs; single median pronotal tubercle present, well-developed (shared with members of the former *Cyphomyrmex strigatus* group, but vestigial in species of the former *Mycetophylax s.s.*); node of petiole with a pair of tubercles or spines; gastral tergite I strongly rugose and with small tubercles from each of which arises a single, short, erect hair.

Description. The worker of *Mycetophylax asper* was first described by Emery [26] from a single specimen collected in Argentina. Here, we complement Emery's description of the worker based on the specimen studied by him and additional workers from multiple nest series collected in the municipality of Chapecó, Santa Catarina, Brazil.

Head: in full-face view, and excluding mandibles, as long or slightly longer than wide (HL 0.75–0.83, HW 0.72–0.83, CI 96–105); in full-face view, cephalic margin medially emarginate (Figs 2B, 3G and 4A); in full-face view, preocular carina extending posterad to cephalic corner, meeting frontal carina to form conspicuous antennal scrobes (Figs 2B and 2D; 3A and 3G; 4A and 4D); in lateral view, antennal scrobes reticulate (Fig 4D); in full-face view, supraocular tooth triangular, carina-like (Figs 2B, 3G and 4A); in profile, supraocular tooth carina-like and separated from preocular carina by shallow groove (Fig 4D); in profile, dorsum of head covered with short, simple, erect hairs (best seen in Fig 4D); in full-face view, frontal lobes broadly expanded (FLD 0.52–0.60, RFLDI 70–75, RFLDII 71–76), covering antennal insertions (Figs 2B, 3G and 4A); upper margin of frontal carina with short simple hairs (Fig 4A); dorsum of head with short, erect, simple hairs; dorsum of head rugose (Fig 4A); in full-face view, lateral margin of body of clypeus with pair of frontoclypeal teeth directly underneath frontal lobes (Figs 4A and 5A); anterior margin of clypeal apron smooth and convex, interrupted medially by conspicuous notch from which a short, stout, median clypeal seta arises (Figs 4A and 5A); mandibles long (ML 0.47–0.53, MI 59–68); masticatory margin of mandibles 6–7-toothed, with teeth 3 and 4 (counting from base) slightly smaller than basal teeth 1 and 2, apical tooth largest (Fig 5A); outer margin of mandibles sinuous; dorsum of mandibles striate (Figs 4A and 5A); eyes (EL 0.12–0.13; OI 15–17) convex, with 7–8 ommatidia in longest row (34–42 ommatidia total) (Fig 4D); antennal scapes not surpassing cephalic corner when in repose (SL 0.49–0.59, SI 63–75) (Figs 2B and 2D); apical half of antennal scapes gradually broader than basal half (Figs 2B and 3G); leading margin of antennal scape with broad carina apically (Figs 3G

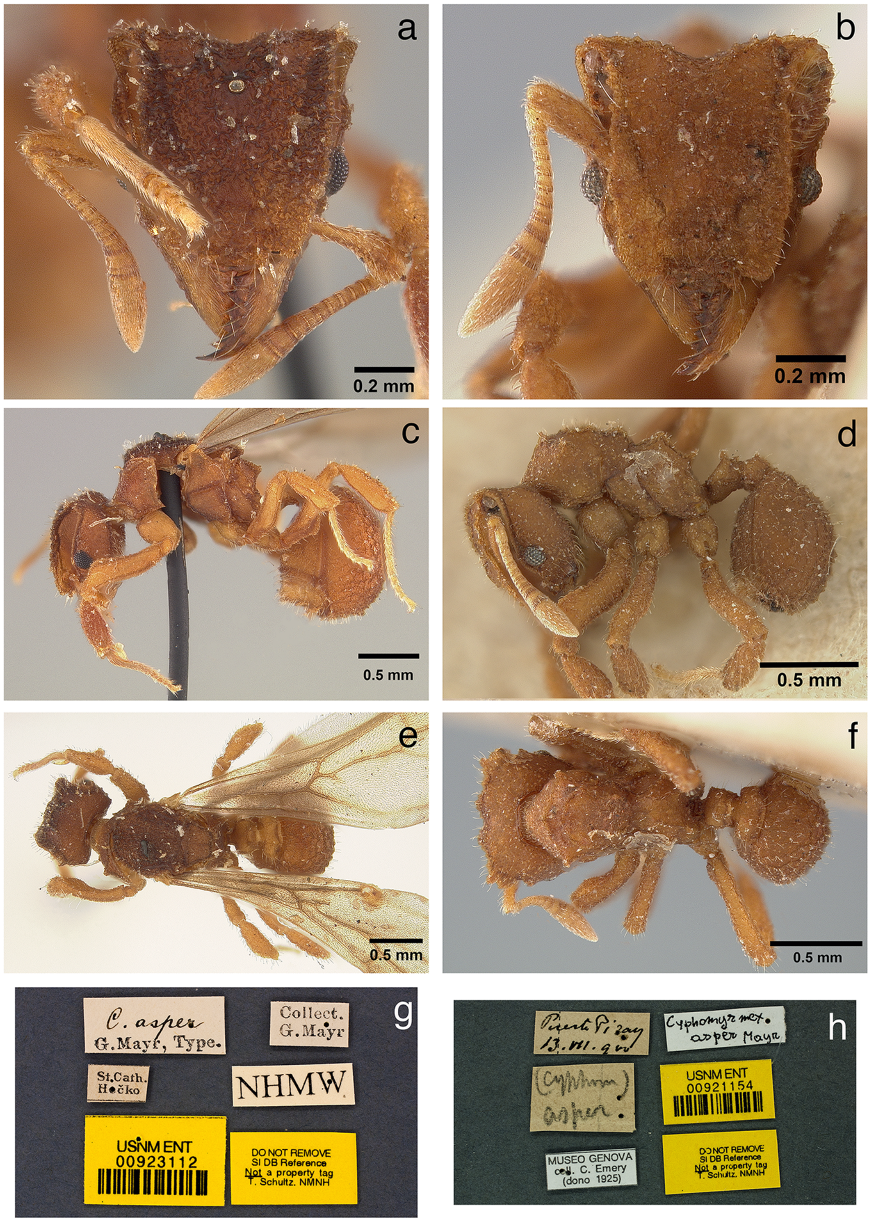

Fig 2. The queen (holotype) and first-described worker of *Mycetophylax asper*. a) Queen, full-face view; b) worker, full-face view; c) queen, lateral profile; d) worker, lateral profile; e) queen, dorsal view; f) worker, dorsal view; g) queen, specimen labels; h) worker, specimen labels.

https://doi.org/10.1371/journal.pone.0176498.g002

and 4A); dorsum of antennal scapes covered with erect, simple hairs, leading margin of scapes with decumbent simple hairs, posterior margin of scapes with appressed simple hairs. Palpal formula 4,2 (Fig 4D).

Mesosoma: In fronto-dorsal and lateral view, pronotum with single median anterior pyramidal tubercle, connected by thin carinae to robust lateral pronotal tubercles, somewhat separating pronotum from mesonotum (Figs 2D and 2F; 3A and 3B; 4G and 4J); dorsum of pronotum rugulose-foveolate (best seen with high magnification, see Fig 4G and 4J); pronotum lacking humeral tubercles; in lateral view, inferior corner of pronotum armed with

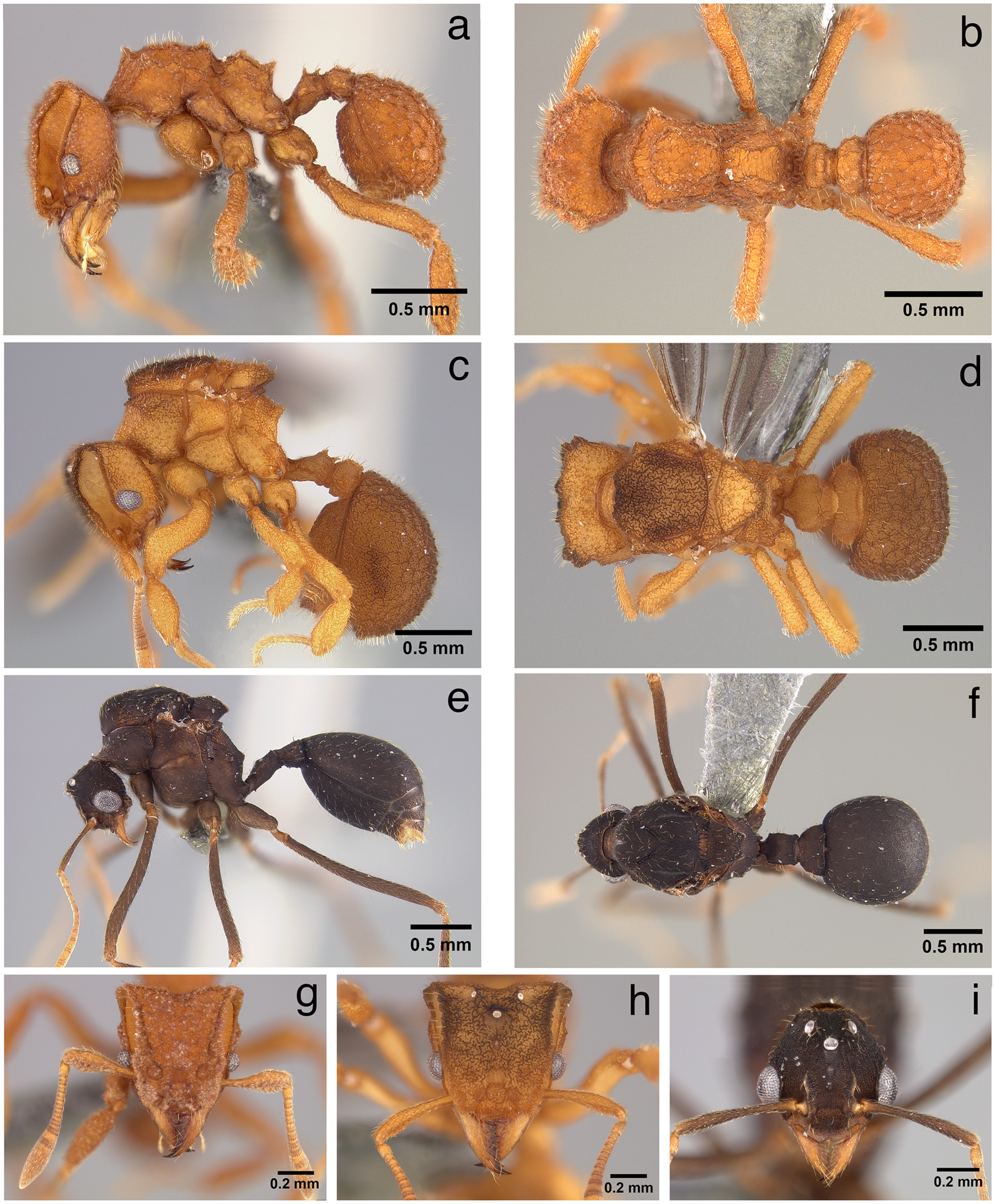

Fig 3. Worker, queen, and male of *Mycetophylax asper*. a) Worker, lateral profile; b) worker, dorsal view; c) queen, lateral profile; d) queen, dorsal view; e) male, lateral profile; f) male, dorsal view; g) worker, full-face view; h) queen, full-face view; i) male, full-face view.

https://doi.org/10.1371/journal.pone.0176498.g003

triangular tooth (Figs 2D, 3A and 4G); in ventral view, propleural plates whitish, almost certainly due to the presence of actinomycete bacteria ([76]; Fig 5C). In dorsal view, lateral mesonotal tubercles carina-like, connected anteriorly by thin transverse carina (Figs 2F, 3B and 4J),

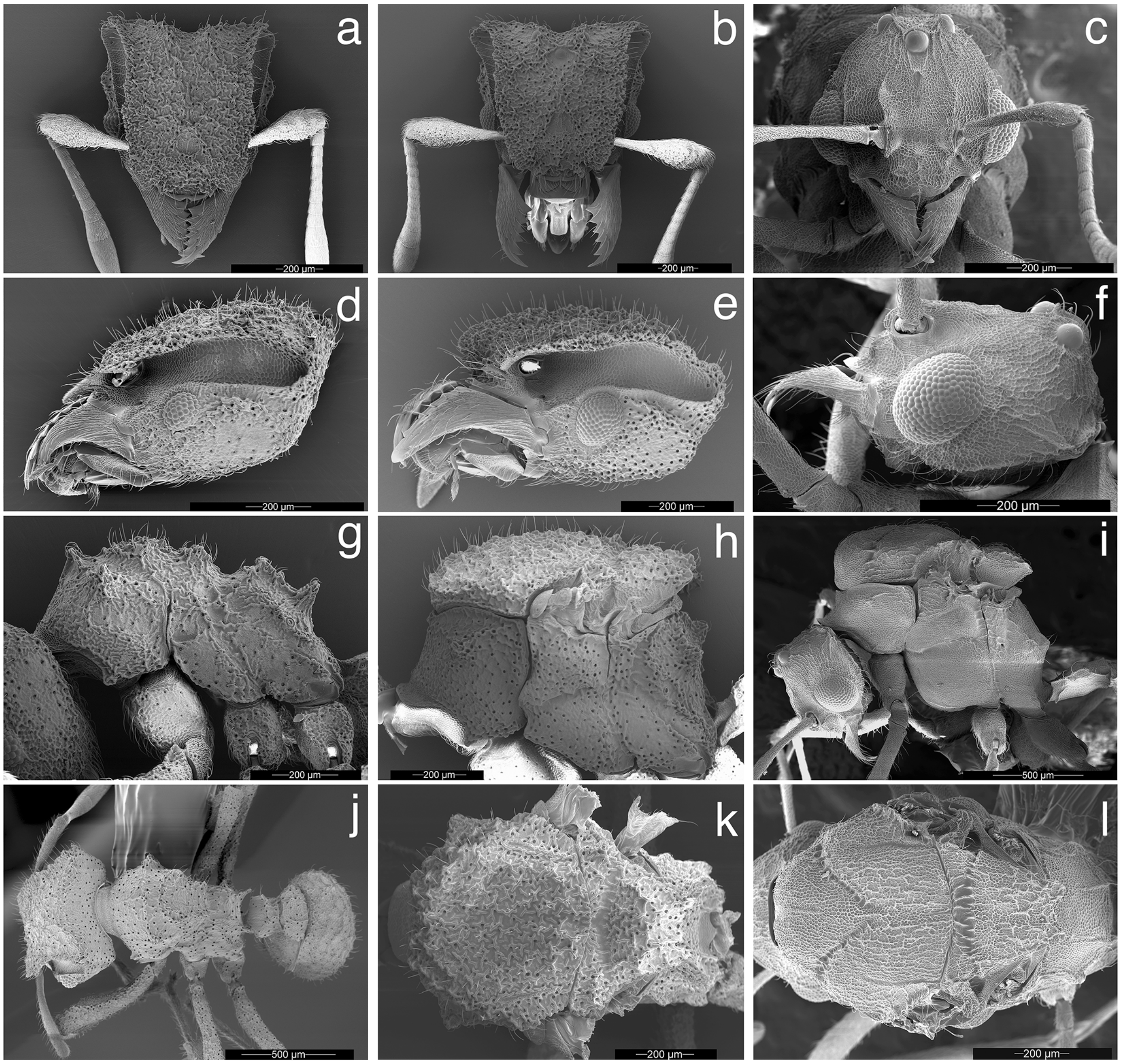

Fig 4. Worker, queen, and male of *Mycetophylax asper*, SEM images. a) Worker, full-face view; b) queen, full-face view; c) male, full-face view; d) worker head, lateral view; e) queen head, lateral view; f) male head, lateral view; g) worker mesosoma, lateral profile; h) queen mesosoma, lateral profile; i) male mesosoma, lateral profile; j) worker, dorsal view; h) queen, dorsal view; i) male, dorsal view.

https://doi.org/10.1371/journal.pone.0176498.g004

anterior mesonotal margin broadly convex; in dorsal view, area circumscribed by lateral and posterior mesonotal tubercles slightly concave and weakly sculptured; in lateral view, posterior mesonotal tubercles triangular (Fig 4G); in lateral view, mesopleural margin (katepisternum) with thin but conspicuous carina (Fig 3A); metanotal groove deep. In lateral view, anterior portion of propodeum with pair of small tubercles (Figs 2D, 3A and 4G); posterior propodeal tubercles larger and acute (Figs 2D, 3A and 4G); anterior and posterior propodeal tubercles connected by lateral carinae; declivity of propodeum shorter than base of propodeum and lacking lateral carinae; in dorsal view, lateral face of propodeum with conspicuous carina, arising anterior to propodeal spiracle to form small but conspicuous tubercle; second, smaller, rounded tubercle arising posterior of spiracle, near the margin of metapleural gland bulla;

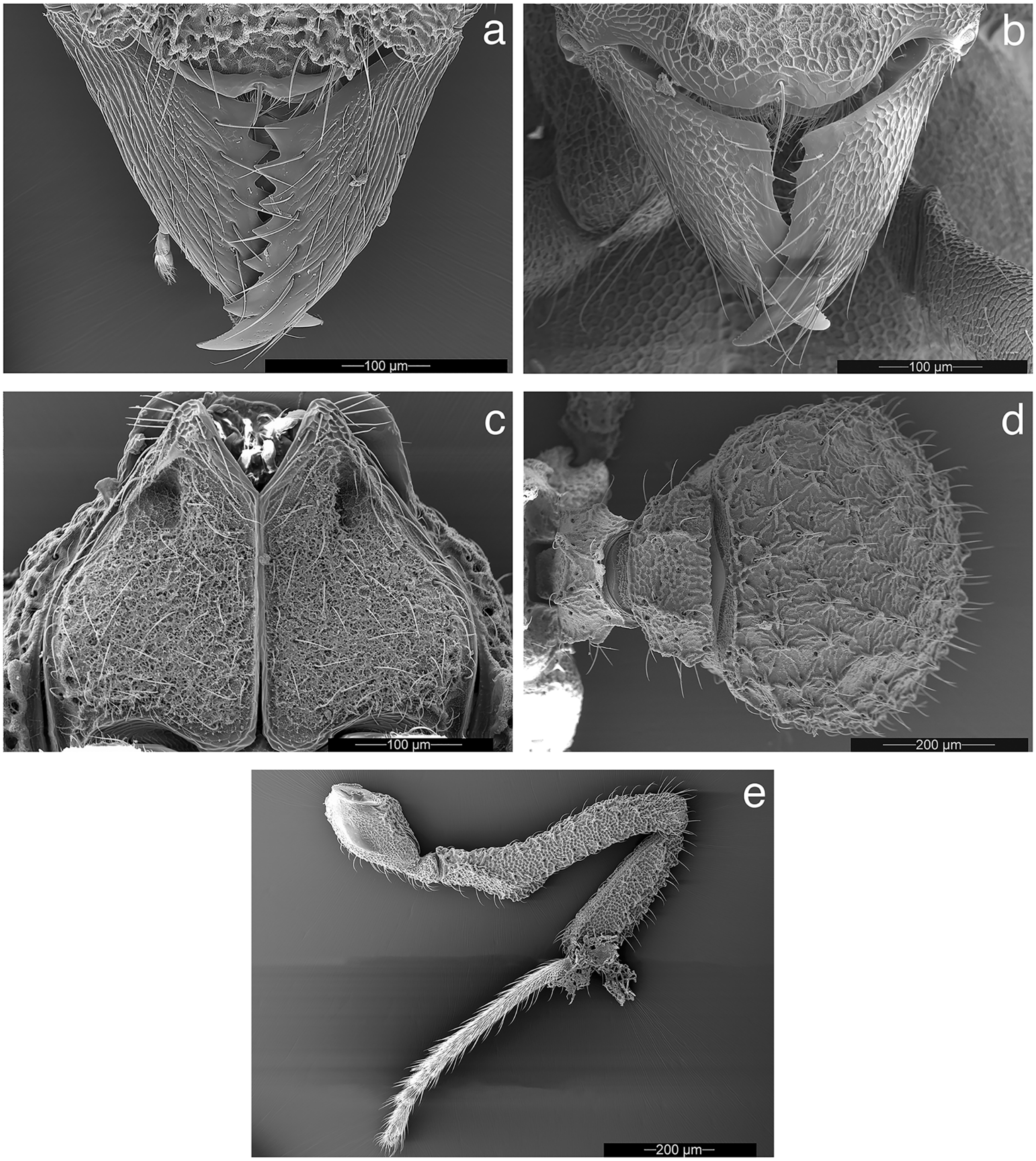

Fig 5. Morphological details of *Mycetophylax asper*, SEM images. a) Worker, mandibles; b) queen, mandibles; c) worker, propleural plates; d) worker, dorsal view of petiole, postpetiole, and gastral tergite I; e) worker, hind leg showing the ventral femoral carina.

https://doi.org/10.1371/journal.pone.0176498.g005

propodeal lobes vestigial to absent. Hind femur and, to a lesser extent, mid femur with pair of conspicuous ventral carinae, produced in the basal one-third into strong ventro-posterior lobe (Fig 5E). Outer margin of mid and hind tibia with short, erect hairs (Fig 5E).

Metasoma: In lateral view, peduncle of petiole vestigial, with small antero-ventral tooth (Figs 2D and 3A); ventral margin of petiole sinuous (Figs 2D and 3A); in dorsal view, antero-

dorsal portion of petiole flattened (Figs 2F and 3B); node of petiole with anterior pair of tubercles rounded at tip and posterior margin of petiolar node with strong transverse carina, giving the impression of posterior tubercle in lateral view (Figs 2D and 3A). In dorsal view, petiole somewhat subquadrate, anterior angles tooth-like (Figs 2F and 3B); lateral portion of petiole with short, erect, simple hairs. In dorsal view, postpetiole wider than long (PPL 0.15–0.17, PPW 0.32–0.34, PPI 196–222), broadly convex anteriorly (Figs 2F and3B). Dorsum of postpetiole with pair of carinae (Figs 2F and 3B); area demarcated by carinae weakly concave medially. Gastral tergite I strongly rugulose and with small tubercles, from each of which arises a single, short, erect hair (Figs 2D and 2F; 3A and 3B; 4J; 5D).

Color: Individuals mostly yellowish to ferrugineous in color. Integument matte, rugose, and foveolate (Figs 2B, 2D and 2F; 3A, 3B and 3G; 4A, 4D, 4G and 4J; 5D).

Measurements. WORKER (specimen from Argentina [26]). EL (0.13) 0.12–0.13, FLD (0.58) 0.52–0.60, GL (0.79) 0.71–0.81, HFL (0.76) 0.71–0.81, HL (0.80) 0.75–0.83, HTL (0.46) 0.42–0.48, HW (0.79) 0.72–0.83, ML (0.48) 0.47–0.53, MSL (0.05) 0.05, PL (0.34) 0.28–0.40, PPL (0.17) 0.15–0.17, PPW (0.32) 0.32–0.34, PW (0.56) 0.51–0.59, SL (0.58) 0.49–0.59, TL (3.58) 3.34–3.74, WL (1.01) 0.97–1.10, CI (98) 96–105, MI (59) 59–68, MSI (11) 6–11, OI (17) 15–17, PPI (196) 203–222, RFLDI (73) 70–75, RFLDII (74) 71–76, SI (73) 63–75 (n = 12).

Queen: Similar to the worker with modifications expected for the caste and with the following differences:

Head: Dorsum of head densely rugulose, dark brown in color (Figs 2A, 3H and 4B). Anterior ocellus embedded in deep pit, carinate posteriorly (Figs 2A, 3H and 4B). A pair of carinae originating at level of posterior ocelli and extending posterad to cephalic margin (Figs 2A, 3H and 4B).

Mesosoma: In dorsal view, anterior portion of pronotum with weakly impressed rugae (Figs 2C, 3C and 4H). Median pronotal tubercle absent (Figs 2C, 3C and 4H); lateral pronotal tubercles connected by a conspicuous carina, forming an obtuse angle at the midline, i.e., where the median pronotal tubercle occurs in the worker (Figs 2C, 3C and 4H). Dorsum of mesoscutum densely rugulose (Figs 2E, 3D and 4K); parapsidal lines present, discrete (Figs 2E, 3D and 4K); parascutal lobes with irregular lateral carinae (Figs 2E, 3D and 4K). Scutellum lacking lateral projections (Figs 2E, 3D and 4K); posterior margin of scutellum lacking tubercles, straight or slightly concave (Figs 2E, 3D and 4K). Base of propodeum with small but conspicuous lateral carinae (Fig 4H); declivity of propodeum lacking lateral carinae (Fig 4H); propodeal tubercles present, triangular; base of propodeum shorter than declivity of propodeum (Figs 2E, 3D and 4K).

Color: Head and body mostly yellowish to ferrugineous; dorsum of head, mesosoma, and gaster tend to be darker in color. Pilosity as in the worker.

Wings smoky, covered with minute pilosity. Forewing lacking pterostigma and with five closed cells present. Hindwing with reduced venation, a single closed cell present (Fig 6A).

Measurements. QUEEN (Holotype). EL (0.18) 0.17–0.19, FLD (0.73) 0.65–0.74, GL (1.18) 1.13–1.28, HFL (0.91) 0.84–0.94, HL (0.90) 0.82–0.93, HTL (0.56) 0.47–0.57, HW (0.91) 0.88–0.98, ML (0.60) 0.53–0.61, MSL (0.05) 0.03–0.05, PL (0.48) 0.36–0.49, PPL (0.17) 0.15–0.22, PPW (0.53) 0.50–0.54, PW (N/A) 0.75–0.85, SL (0.65) 0.61–0.68, TL (4.72) 4.26–4.76, WL (1.39) 1.20–1.34, CI (101) 102–109, MI (67) 58–70, MSI (9) 4–9, OI (20) 19–20, PPI (308) 230–359, RFLDI (81) 78–82, RFLDII (79) 73–77, SI (72) 65–77 (n = 17).

Male: Head: excluding eyes, slightly longer than wide (HL 0.53–0.65, HW 0.49–0.60, CI 98–100); in full-face view, posterior cephalic margin strongly convex (Figs 3I and 4C); preocular carina present, extending posterad almost to level of anterior ocellus, failing to reach cephalic margin (Figs 3I and 4C); dorsum of head (frons) areolate with torulose interspace sculpture (Fig 4C); median carina absent (Fig 4C). Mandibles triangular, inner margin 5- to 6-toothed, increasing in size towards apex (Figs 4C and 5B); dorsum of mandibles imbricate

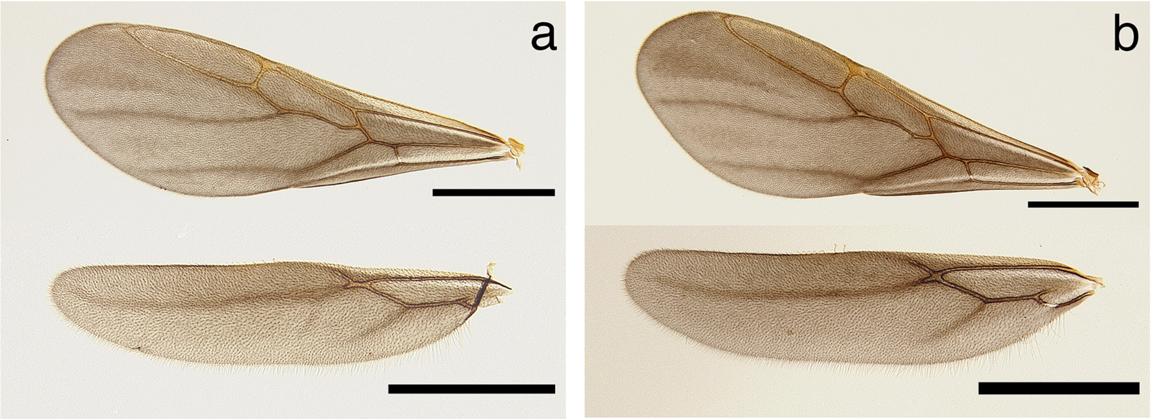

Fig 6. Wings of *Mycetophylax asper*. a) Queen, fore and hind wings; b) male, fore and hind wings. Scale bars in figures represent 1.0 mm in length.

https://doi.org/10.1371/journal.pone.0176498.g006

and with appressed simple hairs (Fig 5B). Clypeal apron thin, shiny, and strongly notched medially (Fig 5B); dorsum of clypeal apron weakly reticulate (Fig 5B); unpaired median seta long (MSL 0.07–0.09, MSI 11–27), emerging from dorsal portion of clypeal apron well removed posterad from border (Fig 5B). In full-face view, body of clypeus subquadrate, circumscribed by lateral carinae, each of which is produced into a small frontoclypeal tooth, directly underneath each frontal lobe (Fig 4C); median portion of body of clypeus, between frontal lobes, with 1 or 2 minute tubercle(s) or blunt projection(s), best seen when looking from above from a viewpoint in which both the occipital collar and the posterior ocelli are visible; posterior margin of clypeus forming deep groove between frontal lobes (Fig 4C). In full-face view, frontal lobes narrow or vestigial, failing to cover antennal insertions (FLD 0.20–0.26, RFLDI 37–41, RFLDII 40–46) (Fig 4C and 4F); frontal carinae extending posterad to join rugae on either side of anterior ocellus (Fig 4C). Antennal scapes long (SL 0.52–0.63, SI 104–110), longer than length of funicular segments I–III combined; antennal scapes surpassing cephalic margin by ~1/2 of their length (Figs 3E and 3I; 4C); antennal scapes thin, lacking expanded carinae as in worker and queen, attaining maximum width near apex (Fig 3I); dorsum of antennal scapes covered with appressed, simple hairs, becoming decumbent at apex (Figs 3I and 4C); integument of antennal scapes as on frons; antennae 12-segmented. Eyes large (EL 0.22–0.25, OI 40–46), convex. In lateral view, gena with prominent carina that extends from base of mandibles to occipital collar (Fig 4F). In lateral view, hypostoma lacking teeth (Fig 4F). Palp formula 4,2.

Mesosoma: Pronotum lacking humeral tubercles; in dorsal view, lateral tubercles of pronotum reduced to carinae, at most forming obtuse angles (Figs 3E and 3F; 4I); in lateral view and, to a lesser extent, in fronto-dorsal view, a minute tooth present at position of median pronotal tubercle, very close to posterior margin of pronotum (Figs 3E and 4I); antero-inferior margin of pronotum, at most, angulate, not produced into a tubercle or tooth (Figs 3E and 4I). In dorsal view, median sulcus and median line of mesoscutum inconspicuous, visible under high magnification (Fig 4L); notauli present, deep and costulate (Fig 4L); parapsidal lines present (Fig 4L); in dorsal view, mesoscutum rugulose in addition to areolate with torulose interspace sculpture (Fig 4L). Oblique mesopleural sulcus (= anapleural sulcus; [77, 78]) deep, with some costulae, excavating the anepisternum so that its lower edge slightly overhangs the katepisternum (Fig 4I). Mesoscutellum with deep, transversely costate scutoscutellar sulcus (Fig 4l); posterior margin of mesoscutellum bidentate (Fig 4l). In lateral view, propodeum lacking teeth, at

most with small carinae forming obtuse angles (Figs 3E and 4I); base of propodeum larger than declivity of propodeum (Figs 3E and 4I).

Legs very long, length of hind femur longer than mesosoma (HFL 1.25–1.60, WL 1.18–1.39, HFI 106–116); ventral margin of mid and hind femur with erect simple hairs on basal half, apical half lacking hairs or with very short, appressed hairs.

Metasoma: In lateral view, peduncle of petiole cylindrical in appearance and with a minute antero-ventral petiolar process (Fig 3E); petiole dorsoventrally nearly flattened, weakly convex dorsally (Fig 3E and 3F), subquadrate; in dorsal view, node of petiole lacking tubercles, at most a pair of very inconspicuous tumosities (Fig 3F); in dorsal view, lateral margin of petiole with thin lateral carina; dorsum of petiole finely reticulate, lacking hairs (Fig 3F). Postpetiole wider than long (PPL 0.20–0.26, PPW 0.36–0.46, PPI 169–203); in dorsal view, postpetiole dome-shaped (Fig 3F); dorsum of postpetiole lacking hairs, except for a pair located near posterior margin. Dorsum of gastral segment I finely and strongly reticulate (Fig 3F).

Fore and hind wings as in the queen (Fig 6B).

Color: Antennal funicular segments, mandibles, and tarsomeres yellowish to light brown, rest of body dark brown (Fig 3E, 3F and 3I).

Measurements. MALE. EL 0.22–0.25, FLD 0.20–0.26, GL 1.16–1.46, HFL 1.25–1.60, HL 0.53–0.65, HTL 1.04–1.30, HW 0.49–0.60, ML 0.32–0.38, MSL 0.07–0.09, PL 0.35–0.44, PPL 0.20–0.26, PPW 0.36–0.46, PW 0.51–0.66, SL 0.52–0.63, TL 3.76–4.54, WL 1.18–1.39, CI 90–94, MI 59–61, MSI 11–27, OI 40–46, PPI 169–203, RFLDI 37–41, RFLDII 40–46, SI 104–110 (n = 5).

Larva: Description based on SEM study of three prepupal worker larvae from a single nest (AJ141020–02).

Body profile "attoid" *sensu* Wheeler [79] and Wheeler & Wheeler [80], i.e., longitudinally curved, bean-shaped, with ventral profile shorter than dorsal (Fig 7A). Thoracic-abdominal articulation absent, thoracic intersegmental constrictions superficial, deep lateral depressions associated with abdominal spiracles absent, and leg vestiges present and visible as open slits ventrally on thorax. Dorsal and lateral body surfaces without setae (Fig 7A); setae on head and venter mostly simple, a few with multifurcate tips (Fig 7B). Genal lobes apparently absent. Supra-antennal and supraclypeal setae absent, four setae on each gena and two on clypeus. A few papilliform spinules present on head, restricted to clypeus and genae. Labrum monolobate, narrow, and inflated, with two distinctly setiform anterior setae (Fig 7B–7D). Mandibles fleshy and subconical. Spinules on mandibles densely covering entire surface (Fig 7C). Mandible with distinct, undivided apical tooth and with no subapical teeth. Mandibular gnathobases absent. Basal portion of maxilla fused with head capsule and maxillary palp widely removed laterad from galea. Galea remarkably enlarged and covered with denticles (Fig 7C). Maxillary palp digitiform, maxillary accessory palpal sensillum apparently absent (Fig 7C). Two setae between galea and palp (Fig 7C). Labium feebly protruding, lateral sericteral protuberances absent, labial palps reduced to sensilla. Spinules present only on anterior surface of labium dorsal to sericteries. Hypopharyngeal spinules densely distributed and predominantly unidentate (a few two-toothed spinules present (Fig 7D)). Ventral surface of first thoracic segment lacking ventromedian lobe and papilliform spinules, and with only four long, simple hairs on ventral surface. Ventromedian surfaces of first and second thoracic segments bearing multiple multidentate spinules (Fig 7D). Second and third thoracic segments each with six long, simple hairs ventrally. First and second abdominal segments each with a non-lobiform ventromedian protuberance, protuberance on first abdominal segment more pronounced than that on second (Fig 7D). Four long, simple hairs arise ventrally on each side of protuberance on abdominal segment one, and two to three hairs (varying between specimens) arise on each side of protuberance on abdominal segment two (Fig 7D). Abdominal segment three with one or two pairs of setae; ventral setae absent on abdominal segments four to nine. A single pair of setae

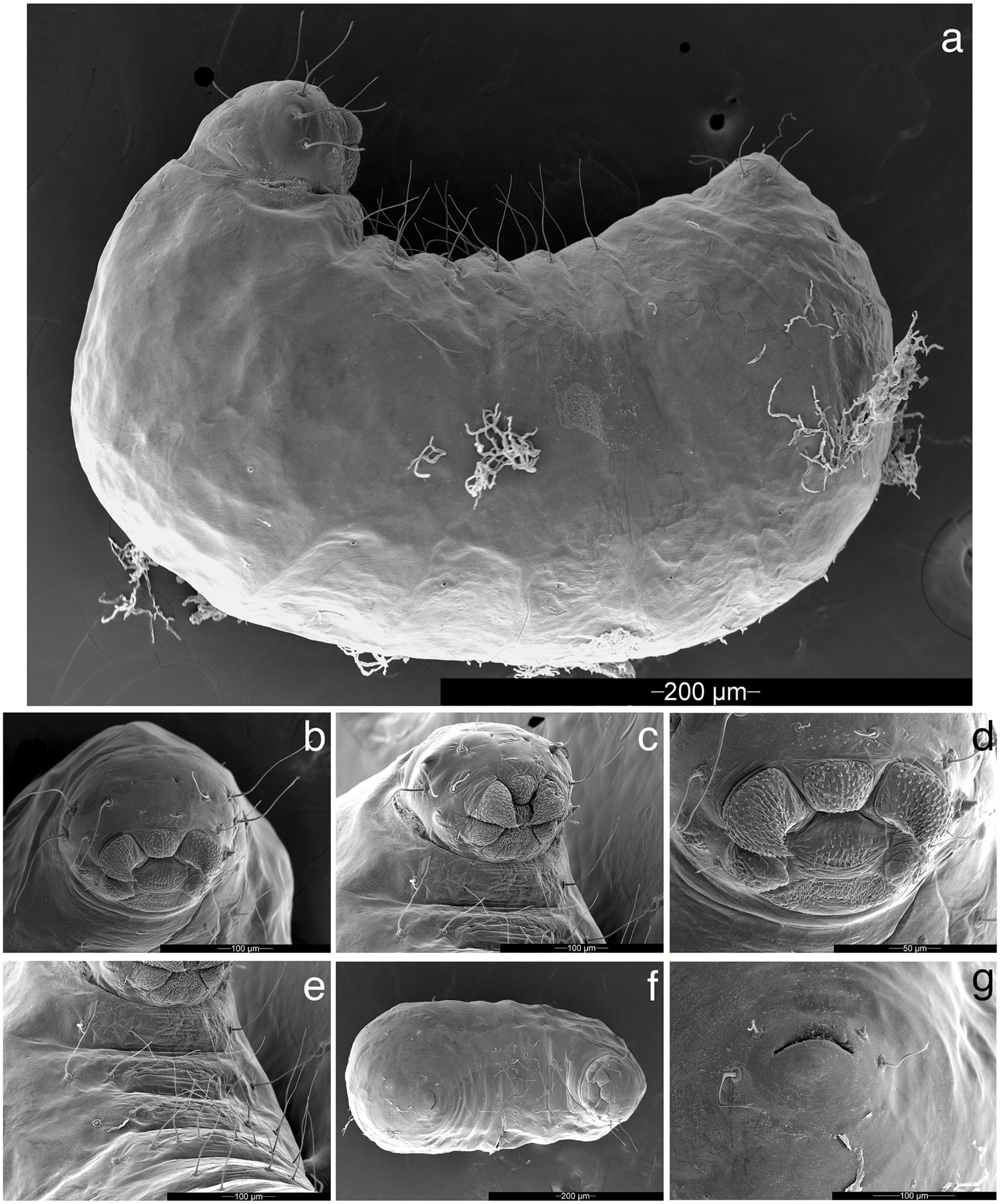

Fig 7. Prepupal worker larva of *Mycetophylax asper*, SEM images. a) Lateral profile; b) and c) head, full-face view; d) mouthparts; e) thorax, ventral view; f) ventral view; g) anal opening (venter at top).

https://doi.org/10.1371/journal.pone.0176498.g007

anterior to anal opening and another pair lateral to opening, all four setae arising on abdominal segment ten (Fig 7F and 7G). Ventral anal lip absent.

Comments. The species *Mycetophylax asper* was first described by Mayr (23) (as *Cyphomyrmex asper*) from a single alate queen collected in Santa Catarina, Brazil. Subsequently, Emery (26) described a worker of *M. asper* collected by F. Silvestri during his travels in southern South America, including Argentina. In his publication, Emery (26) gives the locality of the specimen as Puerto Piramides in Chubut, Argentina. However, the label on the specimen studied by Emery, which is deposited in Genoa, Italy (Fig 2B, 2D, 2F and 2H), indicates the locality "Puerto Piray, 13.vii.900" (Fig 2H). Filippo Silvestri's "Ricordi e itinerary scientifici" (published posthumously in 1959 [81]), which includes the diary of his travels, indicates that Silvestri visited both localities in Argentina: Puerto Pirámides, Chubut Province, southwest of Buenos Aires, at the beginning of December 1899; and Pampa (or Puerto) Piray, Misiones Province, on two occasions, one from 11–13 and another from 19–23 of July, 1900. Both the diary dates and the label data indicate that Silvestri collected the *M. asper* worker specimen in Piray, Misiones, Argentina, rather than in Chubut as erroneously reported in Emery (26) (Maria Tavano, pers. comm.).

Material examined: ARGENTINA: [Provincia de Misiones], Puerto Piray, [26.468686˚ S 54.715880˚ W; elev. 149 m], 13.vii.1900, (*F. Silvestri*) [1w, MSNG, Emery Collection, USNMENTNo.00921154]. **BRAZIL**: Santa Catarina, (*Hečko*) [1aq, NHMW, Mayr Collection, USNMENTNo.00923112]; Santa Catarina, Chapecó, v.1957, (*F. Plaumann*) [1w, MZSP]; Seara, 27.1166667˚ S 052.300˚ W, vi–vii.1999, (*R. da Silva*), Winkler (soil) [2w, MZSP]; Santa Catarina, Chapecó, Floresta Nacional de Chapecó, 27.10306˚ S 52.77898˚ W, elev. 601 m, 18.x.2014, (*A. Jesovnik*, *T. R. Schultz*, & *J. Sosa-Calvo*), nest series, AJ141018–01 [2aq, USNM]; same locality information but AJ141018–04 [1m, USNM]; same locality information but, 27.10307˚ S 52.77904˚ W, elev, 595 m, 20.x.2014, (*J. Sosa-Calvo*, *A. Jesovnik*, & *T. R. Schultz*), nest series, JSC141020–03 [1w, USNM]; same locality information but, (*A. Jesovnik*, *T. R. Schultz*, & *J. Sosa-Calvo*), nest series, AJ141020–02 [1aq CRC; 5aq, USNM]; same locality information but, 21.x.2014, (*J. Sosa-Calvo*, *A. Jesovnik*, & *T. R. Schultz*), nest series, JSC141021–02 [1aq, 1w, DZUP; 1aq, 1m, 2w, USNM]; same locality information but, JSC141021–04 [1m, 1w, CRC; 1w, ICN, 1w MPEG; 1aq, 1m, 1w MZSP; 1w, MBC-UFU; 1aq, 1m, 2w, USNM].

Natural history

Habitat. All but one collection of *Mycetophylax asper* are from the southern state of Santa Catarina, Brazil, including the twenty-one colonies collected by us at the Floresta Nacional de Chapecó (this study). The collections were made from lower to mid elevations (~150–600 m elevation) in remnants of the Atlantic forest in Brazil and, in a single case, in Argentina.

Our field observations indicate that workers of *Mycetophylax asper* forage individually throughout the day for substrate, mainly frass from green-plant-consuming insects. As in many other Attini and fungus-farming ants, workers of *M. asper* feign death when disturbed [13, 82].

Twenty-one nests of *Mycetophylax asper* were excavated at the Floresta Nacional de Chapecó. Information regarding nest architecture and nest demography is summarized in Table 1. These colonies were located in and at the edge of an unpaved dirt road through the forest, covered by grass and apparently rarely used (Fig 8F and 8G).

Nest architecture. Nest entrances of *Mycetophylax asper* consisted of a single hole in the ground (3–4.5 mm in diameter) surrounded by a mound of excavated soil (Fig 8A and 8B). In some cases, the area surrounding the nest entrance was covered by what appeared to be insect frass (Fig 8B). All excavated nests (Table 1) consisted of a single subspherical or dome-shaped (broadly convex on the top and flattened on the bottom) chamber, 1–7 cm in height and 1.5–8 cm in diameter (Fig 8C), located from 9–65 cm below the surface (Table 1). Each chamber contained a compact, small to large fungus garden suspended from the ceiling of the chamber

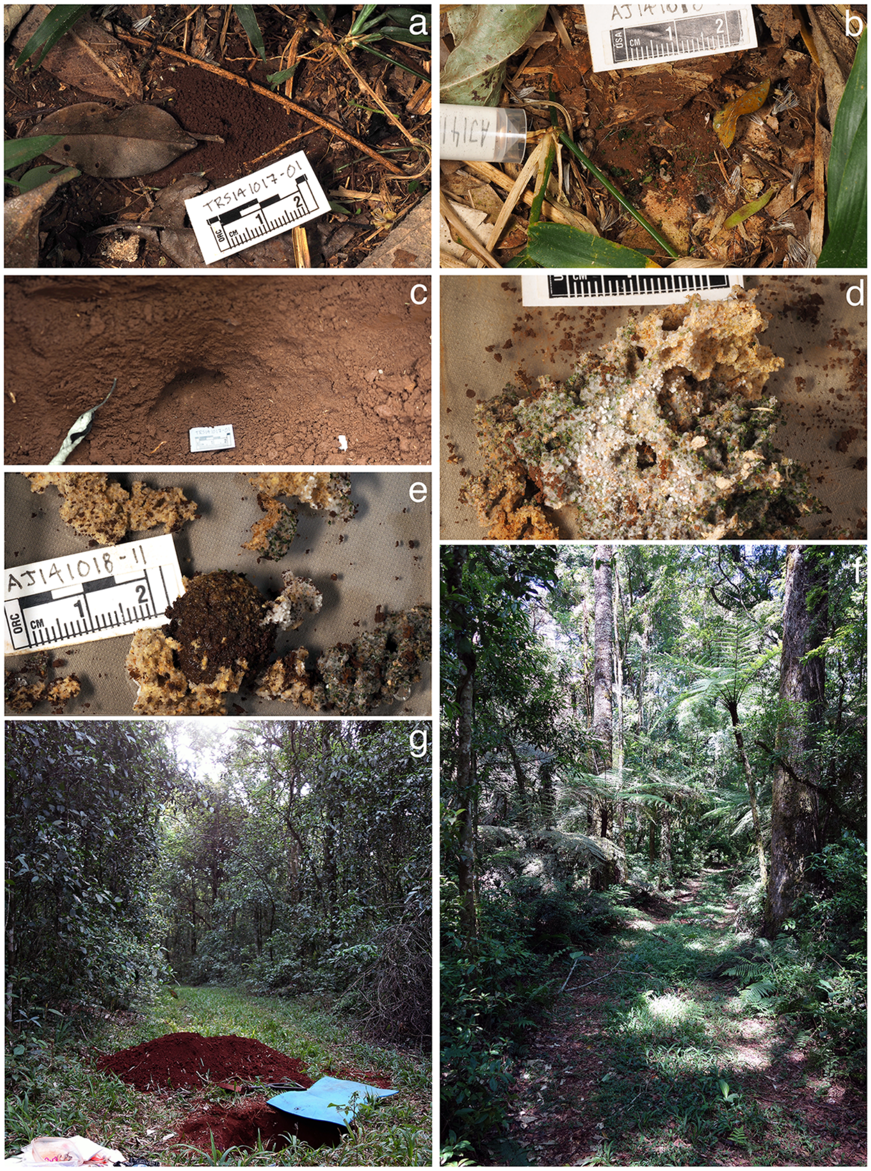

Fig 8. *Mycetophylax asper*, field images. a) Nest entrance, nest TRS141017-01; b) nest entrance, nest AJ141018-11; c) subterranean garden chamber; d) fungus garden and ants in field nest boxes; e) fungus garden and ants in field boxes showing pellet of wet dirt or refuse; g) and f) habitat.

https://doi.org/10.1371/journal.pone.0176498.g008

by rootlets. Most of the fungus gardens extracted from these colonies were of a light yellow color and honeycomb-like in appearance, with some parts of the garden greenish in color due to the presence of newly added substrate (Fig 8D and 8E). In six of the larger colonies, a wet mud pellet was found at the bottom of the chamber, possibly consisting of dirt or refuse (Fig 8E), differing from the infrabuccal pellet piles described by Little et al., [83].

Demography. Colonies of *Mycetophylax asper* are relatively small, containing up to 100 workers. A single, dealate queen was found in every excavated colony, indicating that *M. asper* is monogynous. As of April 2017, eleven of the twenty-one colonies remain alive in artificial nest boxes at the USNM (see Table 1). Observations during a one-year period in the laboratory

suggest that *Mycetophylax asper* produces reproductive forms from January to April, corresponding to the austral summer when temperatures are warmest.

Ant morphology

Morphologically, *Mycetophylax asper* shares with most members of the genus *Mycetophylax*, as defined here, the circumscribed antennal scrobes formed by the joining of the frontal carinae and the posterad-directed preocular carinae at the occipital corners (secondarily reduced in *Mycetophylax conformis* and *Mycetophylax simplex*) (Figs 2B and 2D; 3A and 3G; 4A and 4D), six or more mandibular teeth (Fig 5A), and the presence of a single mid-pronotal tubercle [17, 75], secondarily reduced in the species *M. conformis*, *M. morschi*, and *M. simplex*. In the males of *Mycetophylax asper* the antennal scapes are long (SL 0.52–0.63, SI 104–110), longer than length of funicular segments I–III combined, a condition that is shared with other neoattine species and that contrasts with that of the paleoattines, in which the male antennal scape is shorter than the combined length of funicular segments I–III [19, 35]. In addition, the males of *Mycetophylax asper*, which were unknown at the time of Kempf (17) revision of the *Cyphomyrmex strigatus* group, have 12-segmented antennae, a deviation from the ancestral condition of 13 found in most fungus-farming ants. Although this condition occurs in other members of the former *Cyphomyrmex strigatus*-group (*Mycetophylax faunulus*, *Mycetophylax auritus*) and in *Mycetophylax conformis* [34], it remains unclear whether this shared reduction is due to homology or homoplasy because (i) *Mycetophylax morschi* and *Mycetophylax simplex* males are reported to have 13-segmented antennae [34] and (ii) the 12-segmented condition has arisen independently multiple times in the Attina, including in *Mycetagroicus inflatus*, species of *Sericomyrmex*, *Trachymyrmex opulentus* [23, 34, 36], and some social parasites [84–86].

Based on larval characters, *Mycetophylax asper* is clearly a member of the Neoattina and is more closely related to members of the former *Cyphomyrmex strigatus* group (= *Mycetophylax* s.l.) than to those of the *C. rimosus* (yeast-farming) group. Character states shared with all other fungus-farming ants include the absence of a thoracic-abdominal articulation, the absence of deep lateral depressions associated with the abdominal spiracles, the superficiality of the thoracic intersegmental constrictions, the leg vestiges visible as open slits ventrally on the thorax, the mandibles fleshy and subconical, and, shared with most other attines, the absence of setae on the dorsal and lateral body surfaces (Fig 7A). Character states shared with other neoattines include the fusion of the basal portion of the maxilla with the head capsule combined with the position of the maxillary palp, widely removed laterad from the galea; the labium feebly protruding; the absence of lateral sericteral protuberances; and the reduction of the labial palps to sensilla.

A close relationship of *Mycetophylax asper* to *Mycetophylax* s.l. (i.e., as here redefined) is indicated by the states of no less than six larval characters. First, the presence of non-lobiform ventromedian protuberances on the second and third abdominal segments is similar to the condition observed in the species *Mycetophylax auritus* and *M. faunulus* [87], whereas it differs from the condition in the yeast-cultivating *C. rimosus*-group species in which the first abdominal segment (and, in some species, the second and even third abdominal segments) bears a triangular, lobiform appendage [79, 87]. Second, the hypopharyngeal spinules are predominantly unidentate, a condition previously described only in *M. auritus* and *M. faunulus* [87] (Fig 7D). Third, a single pair of setae arise anterior to the anal opening, another pair arise lateral to the opening, and all four setae arise on abdominal segment ten (Fig 7F and 7G). This pattern is apparently related to the conditions observed in both the *Cyphomyrmex strigatus*- (as formerly defined, = *Mycetophylax* s.l.) and *Cyphomyrmex rimosus*-group species [87], in which there are four ventral setae, but it may also be related to the condition reported in *Mycetophylax*

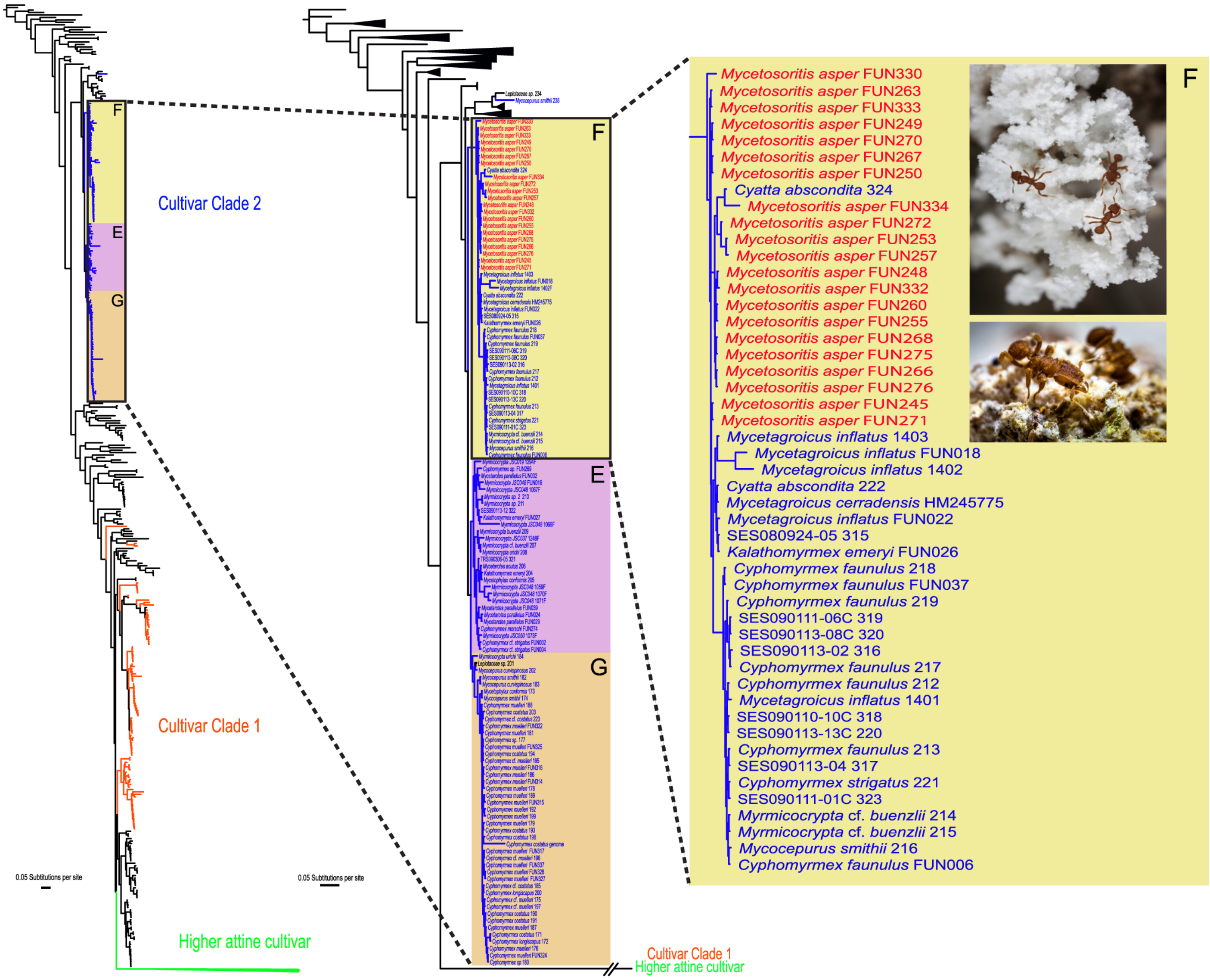

Fig 9. Fungal phylogeny based on Bayesian analysis of ITS sequences. Terminal taxa are named by their ant host species or genera except for free-living Lepiotaceae. Letters F, E, and G refer to subclades of fungal cultivar Clade 2, as defined in Mehdiabadi et al., [41]. Photographs courtesy of: Karolyn Darrow (top) and Don Parsons (bugpix@charter.net) (bottom).

https://doi.org/10.1371/journal.pone.0176498.g009

conformis, in which there are only two ventral setae [87]. Fourth, the ventral anal lip is absent, differing from the condition in most but not all other known *Cyphomyrmex* species, including some members of the newly defined *Mycetophylax*, but shared with *M*. *auritus* and *M*. *conformis*. Fifth, the presence of a distinct mandibular apical tooth is shared with all known *Mycetophylax* s.l. species but not with *C*. *rimosus*-group species, in which the apical tooth is reduced to a spinule. Sixth, the spinules on the head are papilliform as in other known *Mycetophylax s.l.* species, rather than serrate as in most known *C*. *rimosus*-group species.

The larva of *Mycetophylax asper* differs from those of other neoattines in having the galea remarkably enlarged and covered with denticles (Fig 7C). It differs from other *Mycetophylax* species in having the hypopharyngeal spinules densely distributed, an apparent symplesiomorphy shared with most attine ants, including members of the *C*. *rimosus* group. The larva of

Mycetophylax asper is further distinct from all other known *Cyphomyrmex* and *Mycetophylax* species in (i) lacking ventral setae on the ninth abdominal segment and (ii) the apparent absence of genal lobes.

Phylogeny

Results of the ant molecular phylogenetic analyses, which, compared to those of Schultz and Brady [39] and Sosa-Calvo et al., [7], include an additional nuclear gene fragment and additional taxa, place *Mycetophylax asper* in the former *strigatus* group of the genus *Cyphomyrmex* (rendered paraphyletic with respect to *Mycetophylax*) with strong support (Fig 1). Moreover, *Mycetophylax asper* is closely related to the recently described species *Cyphomyrmex andersoni* [75] from Central America (Fig 1), here transferred to *Mycetophylax*. This curious distribution suggests (i) that either or both *Mycetophylax asper* (in southern Brazil) and *M*. *andersoni* (in Mesoamerica) are more widespread than is currently known or (ii) that the distribution of their unknown most recent common ancestor is or was at some time in the past more widespread than either species is today. This pattern of peripherally and disjunctly distributed relict taxa is common across multiple sister clades in the Neoattina and is certainly deserving of further inquiry [14].

Results from the fungal molecular phylogenetic analyses indicate that the fungal species cultivated by *Mycetophylax asper* falls within Clade 2 of the lower attine cultivars (Fig 9, left and center trees) [42, 88]. More specifically, the fungal cultivar of *Mycetophylax asper* belongs to Clade 2, subclade F (Fig 9, center and right trees) of Mehdiabadi et al., [41], likely a single fungal species that is also cultivated by *Cyatta abscondita* [7], *Kalathomyrmex emeryi*, *Mycetagroicus cerradensis* [43], *Mycetagroicus inflatus* [36], *Mycocepurus smithi*, *Myrmicocrypta buenzlii*, and two species in the former *Cyphomyrmex strigatus* group (*Mycetophylax faunulus* and *Mycetophylax strigatus*) [41]. Curiously, the ITS fungal strains most closely related to those cultivated by the forest-dwelling *Mycetophylax asper* are also cultivated by cerrado-dwelling species (*Cyatta abscondita*, *Mycetagroicus inflatus*, *Mycetagroicus cerradensis*, and *Kalathomyrmex emeryi*), in some cases thousands of kilometers away.

Acknowledgments

We are exceedingly grateful to Maria Tavano (MSNG) for facilitating access to the worker described by Emery (1906), for further investigating F. Silvestri's travels in South America, and especially for suggesting that the Chubut locality in Emery (1906) may have been erroneous. We are equally grateful to Rogerio da Silva for invaluable locality information and for directing us onto the right path for locating colonies of *Mycetophylax asper*. We are additionally indebted to Bonnie Blaimer (USNM) for help with translation of Mayr's publication; to ICMbio for granting us permission to collect at Floresta Nacional de Chapecó; to Eugenia Okonski (NMNH) for collections and research support; to Eugenia Okonski and Nick Silverson (NMNH) for help maintaining the live colonies and for helping with the census; and to Scott Whittaker (NMNH) for help with the preparation of the larvae. J.S.-C. is exceedingly grateful to Christian Rabeling for support. T.R.S., and J.S.-C, were partially supported by National Science Foundation (NSF) grants DEB-1456964 and DEB-0949689 and the National Museum of Natural History (NMNH) Small Grants program; T.R.S. by the Smithsonian Institution Scholarly Studies Program; J.S.-C. and A.J. by NMNH Peter Buck Predoctoral Fellowships and Max and Vera Britton Environmental Science Awards (Cosmos Club Foundation); M.B. by the Fundação de Amparo à Pesquisa do Estado de São Paulo (FAPESP; 2011/50226-0) and CNPq (311562/2012-4 and 487639/2012-0); and H.L.V. by the Brazilian Council of Research and

Scientific Development (CNPq grant 302588/2015-9). Fabio S. Nascimento, Rodrigo M. Feitosa, and Lars Vilhelmsen provided many helpful comments that greatly improved the manuscript.

Author Contributions

Conceptualization: JSC TRS AJ.

Data curation: JSC AJ TRS.

Formal analysis: JSC TRS AJ.

Funding acquisition: TRS JSC AJ.

Investigation: JSC AJ HLV MB TRS.

Methodology: JSC AJ TRS.

Project administration: JSC AJ HLV MB TRS.

Resources: JSC AJ HLV MB TRS.

Software: JSC AJ TRS.

Supervision: JSC AJ HLV MB TRS.

Validation: JSC AJ HLV MB TRS.

Writing – original draft: JSC TRS AJ.

Writing – review & editing: JSC AJ HLV MB TRS.

References

1. Zompro O. The Phasmatodea and *Raptophasma* n. gen., Orthoptera incertae sedis Baltic amber (Insecta: Orthoptera). Mitteilungen aus dem Geologisch-Paläontologischen Institut der Universität Hamburg. 2001; 85: 229–61.
2. Klass K- D, Zompro O, Kristensen NP, Adis J. Mantophasmatodea: A new insect order with extant members in the Afrotropics. Science. 2002; 296(5572): 1456–9. https://doi.org/10.1126/science.1069397 PMID: 11964441
3. Klass K- D, Picker MD, Damgaard J, van Noort S, Tojo K. The taxonomy, genitalic morphology, and phylogenetic relationships of Southern African Mantophasmatodea (Insecta). Entomologische Abhandlungen. 2003; 61(1): 3–67.
4. Picker MD, Colville JF, van Noort S. Mantophasmatodea now in South Africa. Science. 2002; 297 (5586): 1475.
5. Cameron SL, Barker SC, Whiting MF. Mitochondrial genomics and the new insect order Mantophasmatodea. Molecular Phylogenetics and Evolution. 2006; 38(1): 274–9. https://doi.org/10.1016/j.ympev.2005.09.020 PMID: 16321547
6. Rabeling C, Brown JM, Verhaagh M. Newly discovered sister lineage sheds light on early ant evolution. Proceedings of the National Academy of Sciences of the United States of America. 2008; 105(39): 14913–7. https://doi.org/10.1073/pnas.0806187105 PMID: 18794530
7. Sosa-Calvo J, Schultz TR, Brandão CRF, Klingenberg C, Feitosa RM, Rabeling C, et al. *Cyatta abscondita*: Taxonomy, evolution, and natural history of a new fungus-farming ant genus from Brazil. PLoS ONE. 2013; 8(11): e80498. https://doi.org/10.1371/journal.pone.0080498 PMID: 24260403
8. Schultz TR, Sosa-Calvo J, Brady SG, Lopes CT, Mueller UG, Bacci M Jr., et al. The most relictual fungus-farming ant species cultivates the most recently evolved and highly domesticated fungal symbiont species. The American Naturalist. 2015; 185(5): 693–703. https://doi.org/10.1086/680501 PMID: 25905511
9. Heath TA, Hedtke SM, Hillis DM. Taxon sampling and the accuracy of phylogenetic analyses. Journal of Systematics and Evolution. 2008; 46(3): 239–57.

10. Soares AER, Schrago CG. The influence of taxon sampling and tree shape on molecular dating: An empirical example from Mammalian mitochondrial genomes. Bioinformatics and Biology Insights. 2012; 6: 129–43. PubMed Central PMCID: PMCPMC3370833. https://doi.org/10.4137/BBI.S9677 PMID: 22693422
11. Ward PS, Brady SG, Fisher BL, Schultz TR. Phylogeny and biogeography of dolichoderine ants: Effects of data partitioning and relict taxa on historical inference. Systematic Biology. 2010; 59(3): 342–62. https://doi.org/10.1093/sysbio/syq012 PMID: 20525640
12. Ward PS, Brady SG, Fisher BL, Schultz TR. The evolution of myrmicine ants: Phylogeny and biogeography of a hyperdiverse ant clade (Hymenoptera: Formicidae). Systematic Entomology. 2015; 40(1): 61–81.
13. Sosa-Calvo J, Jesovnik A, Lopes CT, Rodrigues A, Rabeling C, Bacci M Jr, et al. Biology of the relict fungus-farming ant *Apterostigma megacephala* Lattke, including descriptions of the male, gyne, and larva. Insectes Sociaux. 2017.
14. Branstetter MG, Jesovnik A, Sosa-Calvo J, Lloyd MW, Faircloth BC, Brady SG, et al. Dry habitats were crucibles of domestication in the evolution of agriculture in ants. Proceedings of the Royal Society B. 2017; 284(20170095).
15. Wheeler WM. The fungus-growing ants of North America. Bulletin of the American Museum of Natural History. 1907; 23(31): 669–807.
16. Creighton WS. The ants of North America. Bulletin of the Museum of Comparative Zoolology (Harvard College). 1950; 104: 1–585.
17. Kempf WW. A revision of the Neotropical ants of the genus *Cyphomyrmex* Mayr. Part I. Group of *strigatus* Mayr (Hym. Formicidae). Studia Entomologica. 1964; 7(1–4): 1–44.
18. Kempf WW. Miscellaneous studies on Neotropical ants. VI. (Hymenoptera: Formicidae). Studia Entomologica. 1968; 11(1–4): 369–415.
19. Emery C. Etudes sur les Myrmicinae V. Les genres des Attini; descriptions de nouvelles formes de *Mycocepurus* et de *Myrmicocrypta*. Annales de la Société Entomologique de Belgique. 1913; 57: 250–62.
20. Emery C. Hymenoptera, Fam. Formicidae, Subfam. Myrmicinae. Genera Insectorum. 1922; 174: 207–397.
21. Wheeler WM. Ants of the American Museum Congo expedition. Bulletin of the American Museum of Natural History: New York; 1922.
22. Kusnezov N. El género *Cyphomyrmex* (Hymenoptera, Formicidae) en la Argentina. Acta Zoológica Lilloana (Instituto Miguel Lillo). 1949; 8: 427–56.
23. Mayr GL. Südamerikanische Formiciden. Verhandlungen der Zoologisch-Botanischen Gesellschaft in Wien. 1887; 37: 511–632.
24. MacKay WP. Dos especies nuevas de hormigas de la tribu Attini de Costa Rica y México: *Mycetosoritis vinsoni* y *Mycocepurus curvispinosus* (Hymenoptera: Formicidae). Revista de Biología Tropical. 1998; 46(2): 421–6.
25. Sosa-Calvo J, Brady SG, Schultz TR. The gyne of the enigmatic fungus-farming ant species *Mycetosoritis explicata*. Journal of Hymenoptera Research. 2009; 18(1): 113–20.
26. Emery C. Studi sulle Formiche della fauna Neotropica. XXVI. Formiche raccolte dal prof. F. Silvestri nell'Argentina e nelle regioni limitrofe dell'Uruguay, del Brasile, del Paraguay e del Chile. Bollettino della Societa Entomologica Italiana. 1906; 37: 107–94.
27. Lucas EM, Fortes VB. Frog diversity in the Floresta Nacional de Chapecó, Atlantic Forest of southern Brazil. Biota Neotrópica. 2008; 8(3): 51–61.
28. ICMBio. Plano de Manejo da Floresta Nacional de Chapecó, Santa Catarina. Instituto Chico Mendes de Conservação da Biodiversidade, 2013.
29. Lutinski JA, Mello Garcia FR, Lutinski CJ, Iop S. Diversidade de formigas na Floresta Nacional de Chapecó, Santa Catarina, Brasil. Ciência Rural. 2008; 38(7): 1810–6.
30. Schultz TR. Stalking the wild attine. Notes from Underground. 1993; 8: 7–10.
31. Rabeling C, Verhaagh M, Engels W. Comparative study of nest architecture and colony structure of the fungus-growing ants, *Mycocepurus goeldii* and *M. smithii*. Journal of Insect Science. 2007; 7(40): 1–13.
32. Sosa-Calvo J, Jesovnik A, Okonski E, Schultz TR. Locating, collecting, and maintaining colonies of fungus-farming ants (Hymenoptera: Myrmicinae: Attini). Sociobiology. 2015; 62(2): 300–20.
33. Rabeling C, Cover SP, Johnson RA, Mueller UG. A review of the North American species of the fungus-gardening ant genus *Trachymyrmex* (Hymenoptera: Formicidae). Zootaxa. 2007; 1664: 1–53.
34. Klingenberg C, Brandão CRF. Revision of the fungus-growing ant genera *Mycetophylax* Emery and *Paramycetophylax* Kusnezov rev. stat., and description of *Kalathomyrmex* n. gen. (Formicidae: Myrmicinae: Attini). Zootaxa. 2009; 2052: 1–31.

35. Sosa-Calvo J, Schultz TR. Three remarkable new fungus-growing ant species of the genus *Myrmicocrypta* (Hymenoptera: Formicidae), with a reassessment of the characters that define the genus and its position within the Attini. Annals of the Entomological Society of America. 2010; 103(2): 181–95.
36. Jesovnik A, Sosa-Calvo J, Lopes CT, Vasconcelos HL, Schultz TR. Nest architecture, fungus gardens, queen, males, and larvae of the fungus-growing ant *Mycetagroicus inflatus* Brandão & Mayhé-Nunes. Insectes Sociaux. 2013; 60: 531–42. https://doi.org/10.1007/s00040-013-0320-8 PMID: 24273337
37. Rabeling C, Schultz TR, Bacci M Jr, Bollazzi M. *Acromyrmex charruanus*: a new inquiline social parasite species of leaf-cutting ants. Insectes Sociaux. 2015; 62(3): 335–49.
38. Rabeling C, Sosa-Calvo J, O'Connell LA, Coloma LA, F F. *Lenomyrmex hoelldobleri*: a new ant species discovered in the stomach of the dendrobatid poison frog, *Oophaga sylvatica* (Funkhouser). ZooKeys. 2016; 618: 79–95.
39. Schultz TR, Brady SG. Major evolutionary transitions in ant agriculture. Proceedings of the National Academy of Sciences of the United States of America. 2008; 105(14): 5435–40. https://doi.org/10.1073/pnas.0711024105 PMID: 18362345
40. Sosa-Calvo J. Systematics of the cryptic fungus-farming ant genus Myrmicocrypta Fr. Smith, with the description of a new genus and species of fungus-farming ants (Hymenoptera: Myrmicinae) [Dissertation]. Digital Repository at the University of Maryland: University of Maryland; 2015.
41. Mehdiabadi NJ, Mueller UG, Brady SG, Himler AG, Schultz TR. Symbiont fidelity and the origin of species in fungus-growing ants. Nature Communications. 2012; 3: 840–. https://doi.org/10.1038/ncomms1844 PMID: 22588302
42. Mueller UG, Rehner SA, Schultz TR. The evolution of agriculture in ants. Science. 1998; 281(5385): 2034–8. PMID: 9748164
43. Solomon SE, Lopes CT, Mueller UG, Rodrigues A, Sosa-Calvo J, Schultz TR, et al. Nesting biology and fungiculture of the fungus-growing ant, *Mycetagroicus cerradensis*: New light on the origin of higher-attine agriculture. Journal of Insect Science (Online). 2011; 11(12): 1–14.
44. Maddison WP, Maddison DR. Mesquite 3.0: A modular system for evolutionary analysis. 2015.
45. Katoh K, Kuma K-I, Toh H, Miyata T. MAFFT version 5: Improvement in accuracy of multiple sequence alignment. Nucleic Acids Research. 2005; 33(2): 511–8. https://doi.org/10.1093/nar/gki198 PMID: 15661851
46. Katoh K, Misawa K, Kuma K- I, Miyata T. MAFFT: A novel method for rapid multiple sequence alignment based on Fast Fourier Transform. Nucleic Acids Research. 2002; 30(14): 3059–66. PMID: 12136088
47. Katoh K, Standley DM. MAFFT multiple sequence alignment software version 7: Improvements in performance and usability. Molecular Biology and Evolution. 2013; 30(4): 772–80. PubMed Central PMCID: PMCPMC3603318. https://doi.org/10.1093/molbev/mst010 PMID: 23329690
48. Kearse M, Moir R, Wilson A, Stone-Havas S, Cheung M, Sturrock S, et al. Geneious Basic: an integrated and extendable desktop software platform for the organization and analysis of sequence data. Bioinformatics. 2012; 28(12): 1647–9. https://doi.org/10.1093/bioinformatics/bts199 PMID: 22543367
49. Lanfear R, Calcott B, Ho SYW, Guindon S. PartitionFinder: Combined selection of partitioning schemes and substitution models for phylogenetic analyses. Molecular Biology and Evolution. 2012; 29(6): 1695–701. https://doi.org/10.1093/molbev/mss020 PMID: 22319168
50. Stamatakis A. RAxML-VI-HPC: Maximum Likelihood-based phylogenetic analyses with thousands of taxa and mixed models. Bioinformatics. 2006; 22(21): 2688–90. https://doi.org/10.1093/bioinformatics/btl446 PMID: 16928733
51. Ronquist F, Teslenko M, van der Mark P, Ayres DL, Darling A, Höhna S, et al. MrBayes 3.2: Efficient Bayesian phylogenetic inference and model choice across a large model space. Systematic Biology. 2012; 61(3): 539–42. https://doi.org/10.1093/sysbio/sys029 PMID: 22357727
52. Brown JM, Hedtke SM, Lemmon AR, Lemmon EM. When trees grow too long: Investigating the causes of highly inaccurate Bayesian branch-length estimates. Systematic Biology. 2010; 59(2): 145–61. https://doi.org/10.1093/sysbio/syp081 PMID: 20525627
53. Marshall DC. Cryptic failure of partitioned Bayesian phylogenetic analyses: Lost in the land of long trees. Systematic Biology. 2010; 59(1): 108–17. https://doi.org/10.1093/sysbio/syp080 PMID: 20525623
54. Marshall DC, Simon C, Buckley TR. Accurate branch length estimation in partitioned Bayesian analyses requires accommodation of among-partition rate variation and attention to branch length priors. Systematic Biology. 2006; 55(6): 993–1003. PMID: 17345679
55. Spinks PQ, Shaffer HB. Conflicting mitochondrial and nuclear phylogenies for the widely disjunct *Emys* (Testudines: Emydidae) species complex, and what they tell us about biogeography and hybridization. Systematic Biology. 2009; 58(1): 1–20. https://doi.org/10.1093/sysbio/syp005 PMID: 20525565

56. Rabeling C, Gonzales O, Schultz TR, Bacci M, Garcia MVB, Verhaagh M, et al. Cryptic sexual populations account for genetic diversity and ecological success in a widely distributed, asexual fungus-growing ant. Proceedings of the National Academy of Sciences. 2011; 108(30): 12366–71.
57. Rambaut A, Drummond AJ. Tracer 1.5.0. University of Edinburgh, Edinburgh, UK. Available at: http://beast.bio.ed.ac.uk/Tracer2007.
58. Newton MA, Raftery AE. Approximate Bayesian inference with the weighted likelihood bootstrap. Journal of the Royal Statistical Society Series B (Methodological). 1994: 3–48.
59. Suchard MA, Weiss RE, Sinsheimer JS. Bayesian selection of continuous-time Markov chain evolutionary models. Molecular Biology and Evolution. 2001; 18(6): 1001–13. PMID: 11371589
60. Frandsen PB, Calcott B, Mayer C, Lanfear R. Automatic selection of partitioning schemes for phylogenetic analyses using iterative k-means clustering of site rates. BMC Evolutionary Biology. 2015; 15(1): 1–17.
61. ICZN. International code of zoological nomenclature adopted by the International Union of Biological Sciences: International Trust for Zoological Nomenclature London; 1999. 306 p.
62. Wheeler GC. Myrmecological Orthoepy and Onomatology. Grand Forks, North Dakota: University of North Dakota Press; 1956.
63. Oxford Dictionaries. Oxford University Press; 2012. Oxford Latin Dictionary.
64. Oxford University Press; 1996. A Greek-English Lexicon.
65. Mayr GL. in: Radoszkowsky, O. 1884. Fourmis de Cayenne Française. Trudy Russkago Entomologicheskago Obshchestva. 1884; 18: 31–8.
66. Emery C. Formiche della provincia di Rio Grande do Sûl nel Brasile, raccolte dal dott. Hermann von Ihering. Bullettino della Società Entomologica Italiana. 1888; 19: 352–66.
67. Forel A. Note sur les Attini. Annales de la Société Entomologique de Belgique. 1893; 37: 586–607.
68. Emery C. Studi sulle Formiche della fauna Neotropica. Bollettino della Societa Entomologica Italiana. 1894; 26: 137–241.
69. Forel A. Ameisen des Herrn Prof. v. Ihering aus Brasilien (Sao Paulo usw.) nebst einigen anderen aus Südamerika und Afrika (Hym.). Deutsche Entomologische Zeitschrift. 1911: 285–312.
70. Santschi F. Description de quelques nouvelles fourmis de la République Argentine. Anales de la Sociedad Cientifica Argentina. 1917; 84: 277–83.
71. Wheeler WM. Neotropical ants in the collections of the Royal Museum of Stockholm. Part I. Arkiv for Zoologi. 1925; 17(A8): 1–55.
72. Santschi F. Fourmis de la République Argentine, en particulier du territoire de Misiones. Anales de la Sociedad Científica Argentina. 1933; 116: 105–24.
73. Kusnezov N. Nuevas especies de hormigas (Hymenoptera, Formicidae). Revista de la Sociedad Uruguaya de Entomología. 1957; 2: 7–18.
74. Kempf WW. Miscellaneous studies on Neotropical ants. II. Studia Entomologica. 1962; 5: 1–38.
75. Mackay WP, Serna F. Two new species of the *strigatus* species complex of the ant genus *Cyphomyrmex* (Hymenoptera: Formicidae) from Costa Rica and Panama. Journal of Hymenoptera Research. 2010; 19(1): 44–50.
76. Currie CR, Poulsen M, Mendenhall J, Boomsma JJ, Billen J. Coevolved crypts and exocrine glands support mutualistic bacteria in fungus-growing ants. Science. 2006; 311(5757): 81–3. https://doi.org/10.1126/science.1119744 PMID: 16400148
77. Boudinot BE. Contributions to the knowledge of Formicidae (Hymenoptera, Aculeata): a new diagnosis of the family, the first global male-based key to subfamilies, and a treatment of early branching lineages. European Journal of Taxonomy. 2015; 120: 1–62.
78. Yoshimura M, Fisher BL. A revision of male ants of the Malagasy region (Hymenoptera: Formicidae): Key to subfamilies and treatment of the genera of Ponerinae. Zootaxa. 2007; 1654: 21–40.
79. Wheeler GC. The larvae of the fungus-growing ants. American Midland Naturalist. 1948; 40(3): 664–89.
80. Wheeler GC, Wheeler J. Ant larvae of the myrmicine tribe Attini: Second supplement (Hymenoptera: Formicidae). Proceedings of the Entomological Society of Washington. 1974; 76: 76–81.
81. Silvestri F, Russo G. Ricordi e itinerari scientifici: Stab. tipografico G. Genovese; 1959. 784 p.
82. Mehdiabadi NJ, Schultz TR. Natural history and phylogeny of the fungus-farming ants (Hymenoptera: Formicidae: Myrmicinae: Attini). Myrmecological News. 2010; 13: 37–55.
83. Little AEF, Murakami T, Mueller UG, Currie CR. The infrabuccal pellet piles of fungus-growing ants. Naturwissenschaften. 2003; 90(12): 558–62. https://doi.org/10.1007/s00114-003-0480-x PMID: 14676952

84. Gallardo A. Notes systématiques et éthologiques sur les fourmis attines de la République Argentine. Anales del Museo Nacional de Historia Natural de Buenos Aires. 1916; 28(125): 317–44.

85. Rabeling C, Bacci M Jr. A new workerless inquiline in the Lower Attini (Hymenoptera: Formicidae), with a discussion of social parasitism in fungus-growing ants. Systematic Entomology. 2010; 35: 379–92.

86. Schultz TR, Bekkevold D, Boomsma JJ. *Acromyrmex insinuator* new species: An incipient social parasite of fungus-growing ants. Insectes Sociaux. 1998; 45(4): 457–71.

87. Schultz TR, Meier R. A phylogenetic analysis of the fungus-growing ants (Hymenoptera: Formicidae: Attini) based on morphological characters of the larvae. Systematic Entomology. 1995; 20(4): 337–70.

88. Chapela IH, Rehner SA, Schultz TR, Mueller UG. Evolutionary history of the symbiosis between fungus-growing ants and their fungi. Science. 1994; 266(5191): 1691–4. https://doi.org/10.1126/science.266.5191.1691 PMID: 17775630
